# Analysis of Multitrophic Biodiversity Patterns in the Irtysh River Basin Based on eDNA Metabarcoding

**DOI:** 10.3390/biology14121661

**Published:** 2025-11-24

**Authors:** Ye Chen, Tianjian Song, Yuna Zhang, Fangze Zi, Yuxin Huang, Lei Fang, Yu Liu, Hongyang Zhou, Jiang Chang

**Affiliations:** 1 State Key Laboratory of Environmental Criteria and Risk Assessment, Chinese Research Academy of Environmental Sciences, Beijing 100012, China; 19505568068@163.com (Y.C.); 202131470020@mail.bnu.edu.cn (T.S.); 220220932060@lzu.edu.cn (Y.Z.); hyx573542801@126.com (Y.H.);; 2 College of Life Sciences, Anhui Normal University, Wuhu 241000, China; 3 College of Water Sciences, Beijing Normal University, Beijing 100875, China; 4 College of Ecology, Lanzhou University, Lanzhou 730000, China; 5 College of Material Science and Engineering, Beijing University of Chemical Technology, Beijing 100029, China; 2024400189@buct.edu.cn; 6 Chongqing Academy of Ecology and Environmental Sciences, Chongqing 401336, China; zhouhyrainbow@163.com

**Keywords:** environmental DNA, non-native species, plankton, freshwater, aquatic biodiversity, α-diversity, community structure, environmental filtering

## Abstract

In freshwater ecosystems, cross-trophic interactions among biological communities underpin ecosystem stability and functionality. In arid and semi-arid rivers, however, hydrological fluctuations, invasive species, and other perturbations exacerbate the complexity of biological processes. To systematically assess the community structure of fish, eukaryotic plankton, and prokaryotic microorganism in the Irtysh River basin, this study employed environmental DNA (eDNA) metabarcoding for monitoring. High-throughput sequencing of taxa within the study area was conducted via eDNA metabarcoding, coupled with random forest and linear mixed models to dissect the effects of community structure. The eDNA approach effectively unraveled spatial patterns of biodiversity and identified taxon-specific diversity hotspots: invasive fish exerted a facilitative effect on algae and suppressed the richness of protozoa, fungi, and heterotrophic microorganisms, yet had minimal impact on the dominant structure of autotrophic microorganisms. These findings provide a scientific basis for basin-scale ecological management, emphasizing the necessity of balancing habitat preservation and invasive-species control to safeguard ecosystem functionality.

## Simple Summary

In the Irtysh River basin, this study employed eDNA metabarcoding to monitor fish, planktonic organisms, and prokaryotic microorganisms, thereby validating the effectiveness of this approach in assessing biological community diversity within the ecosystem. By analyzing the environmental and biological drivers that shape community diversity, the research clarified the pathways through which invasive fish species impact algal, fungal, and prokaryotic microbial communities. These findings provide a new scientific basis for watershed ecological management, highlighting the critical role of habitat conservation and invasive species control in maintaining ecosystem functions.

## 1. Introduction

Environmental DNA (eDNA) metabarcoding technology, as an innovative monitoring approach, has emerged as a vital tool for aquatic ecosystem monitoring and management. It assesses biodiversity and ecological health by detecting genetic material released by organisms into the environment [1,2]. In contrast to traditional biological monitoring methods, eDNA technology offers advantages such as non-invasiveness, efficiency, high throughput, and relatively low cost. It also enables high-precision taxonomic analysis across multiple groups [3,4] and overcomes the temporal and spatial limitations of traditional sampling in complex water bodies [5]. In recent years, it has been widely applied in aquatic biodiversity assessment, effectively indicating the health of river ecosystems [6,7,8]. When integrated with the multi-species biological integrity index, it facilitates comprehensive ecosystem health evaluation and real-time community dynamics [9,10].

In aquatic ecosystems, biological communities across different levels interact and influence each other, sustaining the stability and functionality of the ecosystem. Microbial communities are highly sensitive to changes in ecological health, particularly in severely disturbed areas, and play a critical role in biogeochemical processes. Their quantitative shifts can rapidly respond to environmental alterations [11,12]. Consequently, microbial indices are widely used in aquatic ecosystem assessments, aiding the development of new theories regarding ecosystem functions [13,14,15,16]. With advancements in eDNA high-throughput sequencing technology, microbial indices have become essential tools for biodiversity conservation, as well as aquatic ecosystem monitoring and management. Recent research has confirmed their effectiveness in assessing aquatic ecosystem health [17]. Phytoplankton and zooplankton form the core of aquatic ecosystems. Environmental changes can quickly alter their functions, causing fluctuations in community structure and diversity [18,19]. Phytoplankton serve as primary producers, while zooplankton regulate their growth, facilitating energy transfer between trophic levels and supporting ecosystem functions [20]. As higher-trophic-level organisms, fish-community structure changes are influenced by environmental factors and can feedback to affect lower-trophic-level communities, thereby impacting material cycling and energy flow within the ecosystem [21].

Meanwhile, the spatio-temporal dynamics of fish and plankton communities in freshwater ecosystems are jointly influenced by multiple factors, including climate, hydromorphology, and human activities. Clarifying their roles and interrelationships is a central issue in basin ecology [13,19]. As primary producers, phytoplankton determine the fundamental supply of substances and energy, while the regulatory roles of zooplankton and heterotrophic microorganisms in energy transfer and decomposition processes are directly linked to the maintenance of ecosystem functions [12,22]. In rivers of arid and semi-arid regions, hydrological fluctuations are pronounced and gradients of nutrients and dissolved oxygen are significant. The spread of non-native fish and aquaculture activities further increase the uncertainty of biological processes. Therefore, it is necessary to systematically assess community composition, α-diversity patterns, and their formation mechanisms within a large-scale, multi-taxon framework. In this study, we employed eDNA metabarcoding in the Irtysh River basin to simultaneously monitor fish, eukaryotic plankton (including protozoa, algae, and fungi), and prokaryotic microorganisms (both autotrophic and heterotrophic). We comprehensively mapped the spatial patterns of α-diversity, compared differences between groups influenced by the presence or absence of non-native fish, and analyzed environmental and biological factors of different α-diversity indices using random forest and linear mixed models. The objective was to provide enhanced explanatory support for cross-trophic-level biodiversity assessment and ecological management of the basin.

## 2. Materials and Methods

### 2.1. Study Area

**Figure 1:**
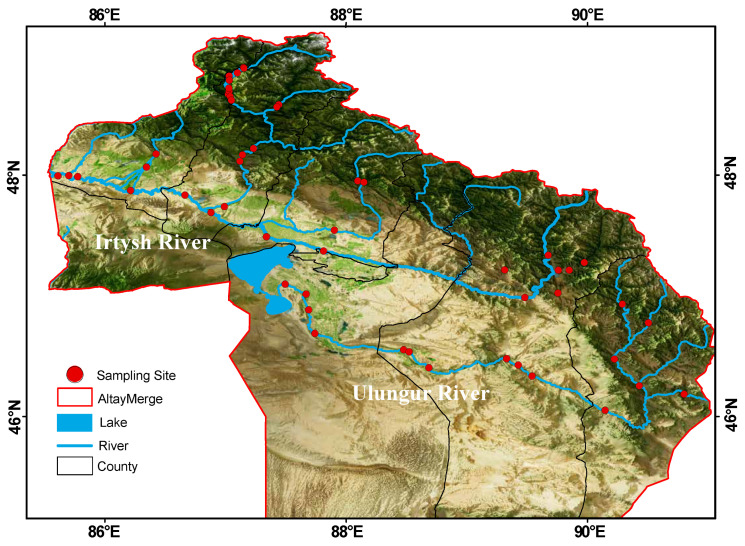
Location of 52 sampling sites in the Irtysh River basin.

The Irtysh River is the only international river originating in China that flows into the Arctic Ocean. It rises from the southern slope of the Altai Mountains in the Xinjiang Uygur Autonomous Region of China, then flows through Kazakhstan and Russia after exiting China at Habahe County, ultimately emptying into the Kara Sea of the Arctic Ocean. With a total length of 4248 km, the river extends 633 km within Chinese territory, draining an area of 57,000 km^2^ and generating an annual runoff of approximately 11.1 billion m^3^. It is the largest river in the Altay region and the second largest in Xinjiang [23]. The Irtysh River and the Ulungur River were originally two independent river systems. However, due to the construction of the “Irtysh River Water Diversion to Ulungur Lake” project during 1986–1987, a 3-kilometer-long canal was dug. In fact, the Ulungur Lake has now become a tributary water body of the Irtysh River [24]. The Irtysh River basin mentioned in this study refers to the part of the Irtysh River within Chinese territory and its main tributaries, as well as the main stream of the Ulungur River and its main tributaries. This study covers 1 city and 6 counties in the Altay region, with a total of 52 sampling sites selected (Figure 1). From basins dominated by agriculture to those almost entirely covered by evergreen coniferous forests, the study encompasses diverse local river environments and land-use patterns.

### 2.2. Sample Collection and Processing

Environmental DNA (eDNA) water samples were collected during a survey conducted in July 2023. Prior to sampling, collection bottles were rinsed three times with ambient water from the sampling sites to minimize contamination. At each site, 1 L of surface water was collected and stored in a car refrigerator or on ice packs to maintain low temperatures. Within 8 h of collection, samples were vacuum-filtered through 50 mm diameter polycarbonate membranes with a 0.22 μm pore size. Between samples, filtration equipment and tools were decontaminated with 10% sodium hypochlorite solution (≈1% available chlorine) and rinsed twice with distilled water to eliminate potential eDNA cross-contamination. Filter membranes were stored in sterile centrifuge tubes at −20 °C until DNA extraction [25,26].

DNA extraction from environmental samples was performed using the DNeasy Blood and Tissue Kit (QIAGEN GmbH, Hilden, Germany). For each sampling site, membranes were extracted independently, and the resulting DNA extracts were pooled to construct a representative eDNA library that integrated data from all replicates. DNA quality was verified via agarose gel electrophoresis, and the extracts were stored at −20 °C for subsequent analyses. The 12S rRNA (fish), 16S rRNA (prokaryotes), and 18S rRNA (eukaryotes) genes were amplified using the following primer pairs: MiFish (MiFish-U-F: GTCGGTAAAACTCGTGCCAGC; MiFish-U-R: CATAGTGGGGTATCTAATCCCAGTTTG) [27]; 515F/806R (515F: GTGYCAGCMGCCGCGGTAA; 806R: GGACTACNVGGGTWTCTAAT) [28]; and 1380F/1510R (1380F: TCCCTGCCHTTTGTACACAC; 1510R: CCTTCYGCAGGTTCACCTAC) [29], respectively. Purified and quantified PCR products were subjected to 2 × 250 bp paired-end high-throughput sequencing on the Illumina PE250 platform (BIOZERON Biotechnology Co., Ltd., Shanghai, China).

### 2.3. Determination of Environmental Factors

Physicochemical parameters of the water, including temperature, pH, electrical conductivity, redox potential, dissolved oxygen, and total dissolved solids, were measured in situ using a YSI Pro Plus multiparameter meter (YSI Incorporated, Yellow Springs, OH, USA). Additionally, 500 mL water samples were collected for laboratory analysis of chemical oxygen demand (COD), total nitrogen (TN), and total phosphorus (TP). All analyses conformed to the Environmental Quality Standards for Surface Water of China (GB 3838-2002, 4th edition) [30]. COD was determined using the dichromate method (Standard number: HJ 828-2017 [31]) with a detection limit of 5 mg/L. TN was analyzed via potassium persulfate digestion–ultraviolet spectrophotometry (Standard number: HJ 636-2012 [32]) with a detection limit of 0.05 mg/L. Ammonium nitrogen (NH_4_^+^-N) and nitrate nitrogen (NO_3_^−^-N) were measured using Nessler’s reagent spectrophotometry (Standard number: HJ 535-2009 [33]; detection limit: 0.025 mg/L) and ion chromatography (Standard number: HJ/T 84-2016 [34]; detection limit: 0.064 mg/L), respectively. TP was determined by continuous flow ammonium molybdate spectrophotometry (Standard number: HJ 670-2013 [35]) with a detection limit of 0.006 mg/L.

### 2.4. Selection of Explanatory Variables and Data Sources

In addition to the physical and chemical properties of water bodies, this study also selected a series of environmental variables that have been proven to affect the distribution of fish and plankton (Table S1), including bioclimate [36], topographic and hydrological conditions [37], soil [38], and the impacts of land use and human activities [39]. All data were obtained from the EarthEnv project [40], which covers almost all spatially continuous variables, specifically for freshwater environments worldwide. This study selected 19 river climate variables, which were created based on the bioclim framework http://worldclim.org/bioclim (accessed on 1 July 2025). It also included 4 hydrological and topographic variables, 8 soil variables, and 12 land-use variables to describe the impacts of sediment erosion and scattered pollution on aquatic organisms. This study also collected the coordinate positions of lakes, reservoirs, and ponds with aquaculture or stock enhancement activities in the Altay region and calculated the straight-line distance from all sampling points to these aquaculture sites. In addition, population density and human footprint index were included in the model as indices of cumulative human activity pressure [41].

### 2.5. eDNA Metabarcoding Analysis

Bioinformatic analyses were conducted using QIIME 2 (version 2024.2) following the workflow described by He et al. [42]. Amplicon sequences were demultiplexed using the demux plugin, and primers were removed with Cutadapt. Sequences were then processed using Vsearch for merging, filtering, chimera removal, FrameBot correction, and singleton exclusion. Operational taxonomic units (OTUs) were clustered from high-quality sequences at a 97% similarity threshold, generating OTU-representative sequences and abundance tables [43].

A non-redundant reference database was constructed to improve taxonomic annotation. Fish 12S rRNA sequences were retrieved from the NCBI Nucleotide (NT) database, the BOLD system, and the mitoFish database (version 3.0) [44] using the CRABS plugin (version 2.0) [45]. Similarly, 18S rRNA sequences of algae, fungi, and protozoa were obtained from the NCBI NT and BOLD (v4). The 16S rRNA gene was annotated against the Greengenes2 database (version 2022.10) [46].

Taxonomic assignment of OTU-representative sequences was performed using the q2-feature-classifier plugin (QIIME 2 2023.9 release) [47] based on similarity thresholds. Community structure and species composition were summarized at the phylum, class, order, family, and genus levels. Species-level annotation followed regional criteria: sequences with >98% similarity to a single species in the database were assigned to that species; sequences with >98% similarity to multiple species were assigned to the lowest common taxonomic rank encompassing all matches [48].

Marine fish and plankton OTUs with no clear geographical distribution pattern were attributed to sewage-derived DNA contamination and excluded from further analyses. All remaining OTUs corresponded to species with known distributions in the study area or to species likely to occur there based on their recorded ranges and physiological traits.

### 2.6. Species Composition and α-Diversity Analysis

α-diversity is a key metric for measuring within-sample species diversity. To complementarily characterize community diversity across three critical dimensions (species richness, individual distribution evenness, and community complexity), this study quantified it using the Shannon–Wiener index [49], the Simpson index [50], Pielou’s evenness index [51], the Chao1 index [52], and the Margalef index [53] via the vegan package in R 4.5.0 [54]. Species accumulation curves were generated using the vegan package 2.6-4. These curves were used to assess the sufficiency of sequencing depth. The taxonomic relationships of fish (order, family, genus) and plankton (phylum, class, order) were visualized using Krona [55].

Random forest models (R package randomForest 4.6-7) were used to explore relationships between the α-diversity metrics of each taxonomic group and explanatory variables, comparing performances across metrics [56]. Linear mixed-effects models (LMMs) were constructed to identify drivers of species richness in fish, prokaryotes, and eukaryotes. Fixed effects included geographical topography, water physicochemistry, anthropogenic activities, bioclimate, land use, soil properties, and biological factors; sampling-site administrative region was included as a random effect to account for differences in aquaculture planning across regions. Explanatory variables were z-score standardized and filtered to exclude those with a variance inflation factor (VIF) of >10 to reduce multicollinearity, ensuring numerical stability and comparable parameter estimates.

All candidate sub-models were compared via full-subset analysis, ranked by corrected Akaike information criterion (AICc), and model averaging was performed on the best-supported models (ΔAICc ≤ 2) to calculate the averaged coefficients for all explanatory variables related to overload [57]. The relative importance of fixed effects associated with overload was expressed as the percentage of explained variance, calculated by dividing the standardized parameter estimate of each overload-related variable by the sum of all standardized estimates in the model [58]. All analyses concerning overload were performed in R using the *randomForest* package 4.6-7, alongside the *glmmTMB* package 1.11.3 (via its glmmTMB function) [59] and the *MuMIn* package 1.48.11 (utilizing its dredge and model.avg functions) [60].

### 2.7. Impact Analysis of Community Structure

From a metacommunity perspective, variation partitioning analysis (VPA) was employed to quantify the relative contributions of environmental filtering and spatial processes to community structure—a common approach in freshwater ecology for identifying metacommunity drivers [61]. Here, VPA explored responses of fish and plankton communities to land use, water environment, hydrological-climatic factors, and anthropogenic factors. Prior to analysis, species abundance data were Hellinger-transformed. To reduce multicollinearity among predictors, principal component analysis (PCA) was applied to 24 geographical-topographic-bioclimatic variables and 19 land use-soil variables, with the first five principal components retained as composite predictors [62,63]. Calculations and visualizations were performed using the *MuMIn* 1.48.11 and *venn* packages 2.1.0 in R 4.5.0.

## 3. Results

### 3.1. Community Composition

**Figure 2:**
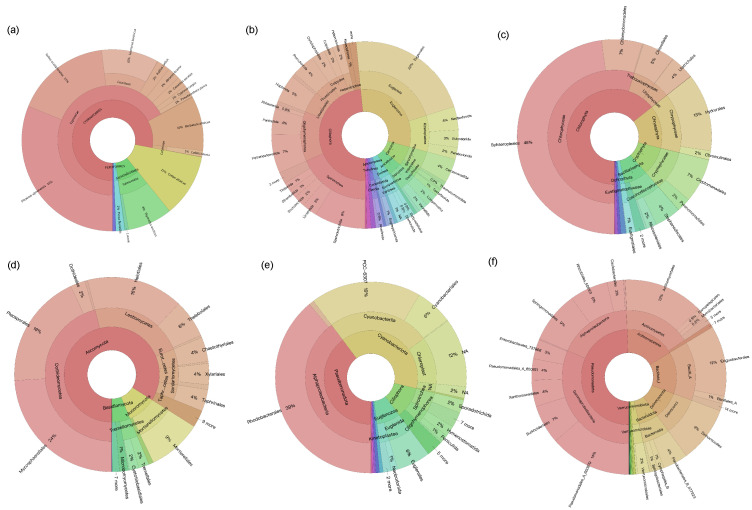
Species composition of the community. (**a**): fish, (**b**): protozoan, (**c**): algal, (**d**): fungi, (**e**): autotrophic, (**f**): heterotrophic.

In this study, two failed amplification sites were removed following high-throughput sequencing analysis. After quality control, removal of singleton sequences and chimeras, and clustering, a dataset comprising 4,041,799 sequences and 143 fish OTUs was obtained from 50 samples. By comparing with historical fish survey records and applying rarefaction normalization, a refined dataset containing 999,383 sequences and 23 OTUs was used for subsequent analyses. A total of 23 fish species, belonging to 10 families and six orders, were detected across the 50 sampling sites, including 9 non-native fish species from three families and two orders: *Misgurnus anguillicaudatus*, *Abbottina rivularis*, *Abramis brama*, *Carassius auratus*, *Cyprinus carpio*, *Hypophthalmichthys nobilis*, *Megalobrama terminalis*, *Pseudorasbora parva*, and *Micropercops swinhonis*. Ranking species by sequence count from highest to lowest, those with more than 100,000 sequences were *Phoxinus ujmonensis* (311,009 reads), *Gobio acutipinnatus* (169,262 reads), *Cottus altaicus* (112,125 reads), and *Leuciscus baicalensis* (101,732 reads). The proportion of each species is shown in Figure 2a.

For other taxa, after quality control, removal of singleton sequences and chimeras, and clustering, datasets were generated from 52 samples (including 5,053,496 sequences and 1680 OTUs for eukaryotic plankton and 6,850,427 sequences and 660 OTUs for prokaryotic microorganisms), and following rarefaction normalization, the datasets used for subsequent analyses consisted of 1,340,748 sequences and 642 OTUs for eukaryotic plankton, and 2,034,646 sequences and 1663 OTUs for prokaryotic microorganisms.

With sequencing depths of 40,120 for fish, 43,163 for eukaryotic plankton, and 39,170 for prokaryotic microorganisms, respectively, the species accumulation curves demonstrated that all biological taxa tended to saturate when the number of samples reached approximately 30–40, indicating that the sampling effort had essentially encompassed the local species diversity (Figure S1).

The protozoan community exhibited high diversity (Figure 2b), with Ciliophora as the most dominant phylum. This phylum includes several class-level groups: Oligohymenophorea, accounting for 9.36%, Spirotrichea 22.61%, Litostomatea 7.10%, and Prostomatea and Colpodea accounting for approximately 4.18% and 0.20%, respectively. Another major group is Euglenozoa, within which Euglenida is the predominant class, accounting for 5.03% of the total protozoan community. Additionally, Kinetoplastea accounts for 5.32%. Rare groups such as Cercozoa and Amoebozoa are also present. The community includes predatory ciliates as well as mixotrophic and heterotrophic euglenoids, reflecting the complexity of the aquatic microfood web.

The algal community is dominated by Chlorophyta, accounting for 64.37% of the total sequences (Figure 2c). Within Chlorophyta, Chlorophyceae and Trebouxiophyceae are the main classes, accounting for 54.82% and 5.50% of the total community, respectively, among which Sphaeropleales in Chlorophyceae is the most abundant order, accounting for 47.88% of the entire algal community. In addition, Chrysophyta accounts for 14.46%, mainly Chrysophyceae, with Hydrurales as the dominant order. There are also smaller proportions of groups such as Cryptophyta and Bacillariophyta. Overall, it is dominated by freshwater green algae, with a certain amount of golden algae, showing typical characteristics of freshwater photosynthetic plankton communities.

The fungi community is dominated by Ascomycota, accounting for approximately 83.97% of the total fungi community (Figure 2d), among which Dothideomycetes is the main class, accounting for 45.97%, followed by Leotiomycetes (22.81%), Eurotiomycetes (4.37%), and Sordariomycetes (5.16%). Basidiomycota accounts for approximately 4.33%, among which Tremellomycetes and Microbotryomycetes are the main groups. In addition, in Mucoromycota, Mucoromycetes accounts for 0.71%. The aquatic fungi community is mainly saprophytic, indicating that it plays an important role in organic matter degradation.

The autotrophic prokaryotic microbial community is mainly composed of Cyanobacteriota, accounting for 49.54% (Figure 2e). It is worth noting that chloroplast groups also account for 14.76%, mainly derived from symbiotic algae or organelle DNA. In Pseudomonadota, Rhodobacterales accounts for approximately 49.36% of the total community, which includes groups such as photoautotrophic and chemoautotrophic photosynthetic bacteria, aerobic and facultative anaerobic chemoheterotrophic microorganisms, and facultative methylotrophic bacteria. Overall, the autotrophic microbial community is dominated by typical freshwater cyanobacteria and Rhodobacteraceae bacteria.

In the heterotrophic prokaryotic microbial community, Pseudomonadota is the main phylum (Figure 2f), accounting for approximately 49.42% of the total heterotrophic community, among which Alphaproteobacteria accounts for 17.95% of the total community and Gammaproteobacteria accounts for 31.47%. Actinomycetota is the second largest group, accounting for 16.99%. Other important groups include Bacillota (0.04%), Deinococcota (9.33%), and Bacteroidota (6.60%). The community is dominated by Gram-negative bacteria involved in organic matter degradation, with a certain proportion of Gram-positive groups, showing typical characteristics of freshwater heterotrophic microorganisms.

### 3.2. Patterns of Alpha Diversity

**Figure 3:**
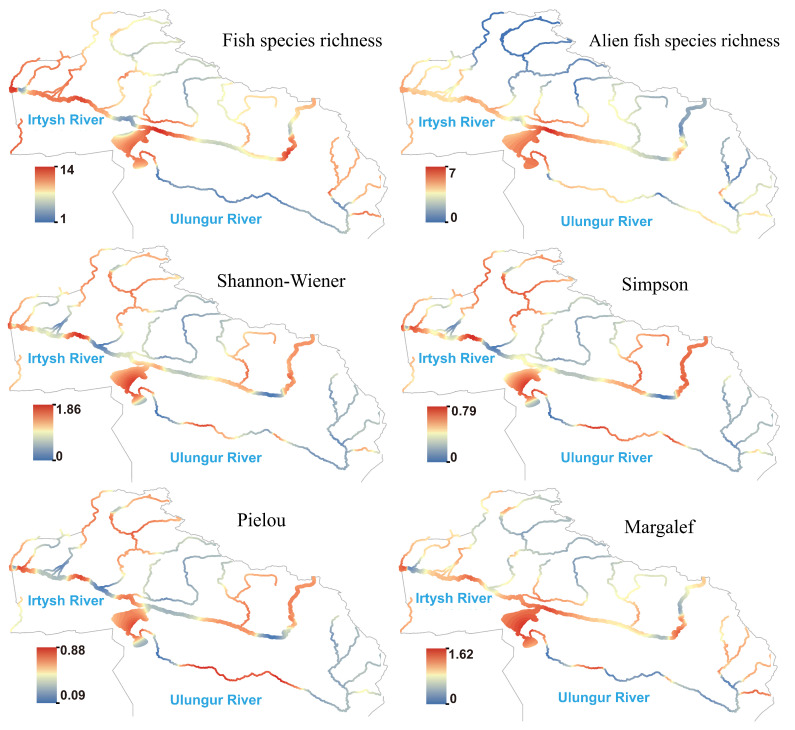
Species richness and α diversity index of fish in the Irtysh River basin.

Based on the eDNA method, the calculated results of α-diversity indices for fish species across 50 sampling sites in 2023 are shown in Figure (Figure 3). The Shannon–Wiener index of fish in the basin ranged from 0 to 1.86; the Simpson index ranged from 0 to 0.79; the Pielou index ranged from 0.09 to 0.88; and the Margalef index ranged from 0 to 1.62. Except for the species richness indices in the downstream being significantly higher than those in the middle reaches, there were no significant differences in other indices among the downstream, middle, and upstream reaches.

**Figure 4:**
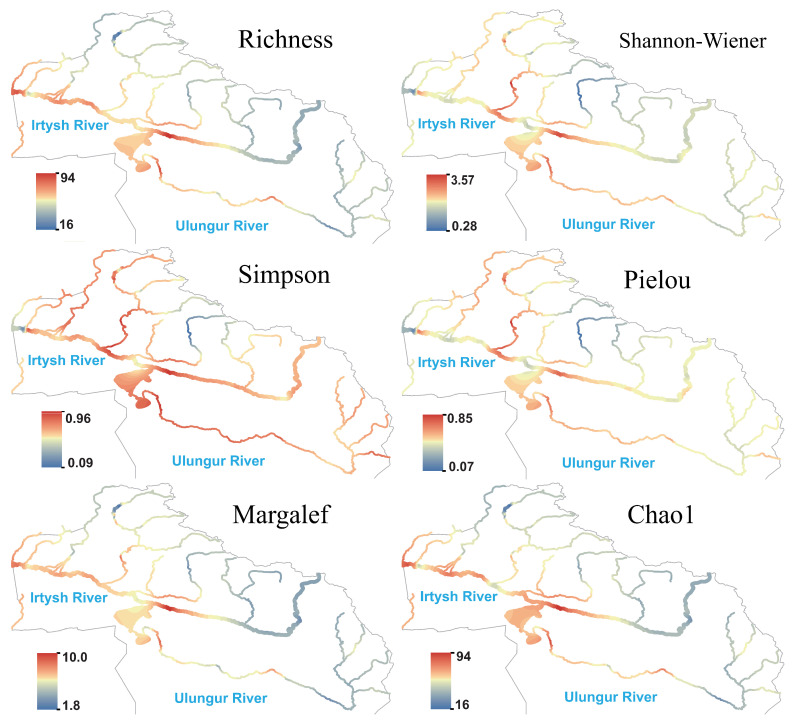
Species richness and α diversity index of algae in the Irtysh River Basin.

Algae formed stable diversity hotspots in the middle and lower reaches of the main streams of the Irtysh River and Ulungur River, with synchronously high values of species richness, Margalef index, and Chao1 index (Figure 4), which gradually decreased from the main stream to the upstream. Hotspots of the Shannon and Pielou indices were located in the middle reaches of the main stream and the Burqin River, indicating high species richness and evenness of algae in these reaches; except for the upper reaches of the Kran River, the Simpson index maintained high levels in the middle and lower reaches of the main stream, reflecting good evenness among dominant algal species.

**Figure 5:**
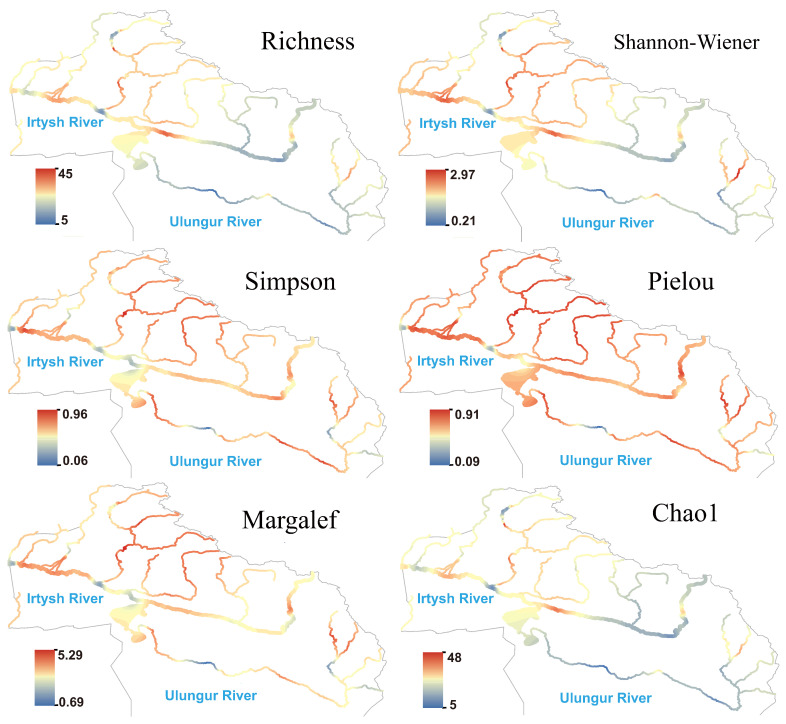
Species richness and α diversity index of protozoa in the Irtysh River basin.

Protozoan generally showed high species richness and Shannon index in the middle and lower reaches of the main stream of the Irtysh River (Figure 5), as well as in the Burqin River and Kran River tributaries, while most reaches of the Ulungur River showed moderate to low values. Hotspots of the Pielou and Simpson indices highly overlapped, indicating that protozoan communities exhibited good evenness in the Irtysh River basin. Compared with algae, the hotspot areas of protozoan expanded more toward the northern high-altitude regions, suggesting their higher sensitivity to local temperature or human activity impacts.

**Figure 6:**
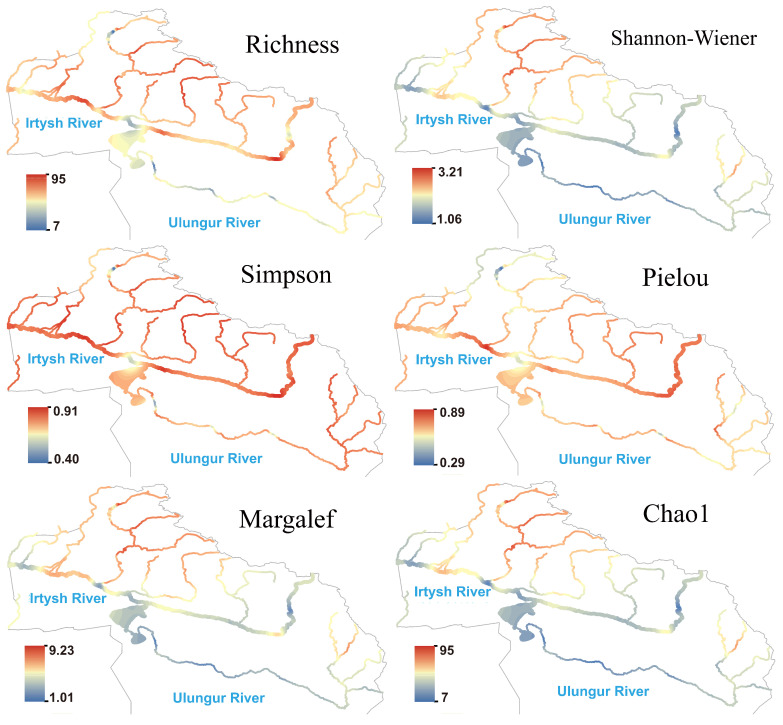
Species richness and α diversity index of fungi in the Irtysh River Basin.

The species richness of fungi showed a pattern of being higher in the Irtysh River and lower in the Ulungur River at the river network scale (Figure 6). The Kanasi River, Hemu River, and Burqin River were high-value regions in terms of the Shannon, Margalef, and Chao1 indices, while some sections of the main stream showed significantly low hotspots, reflecting simple community structures, low species numbers, and absence of strongly dominant species. Both Simpson and Pielou indices were high in the main stream and multiple tributaries, indicating that despite low species numbers in individual regions, community evenness remained good.

**Figure 7:**
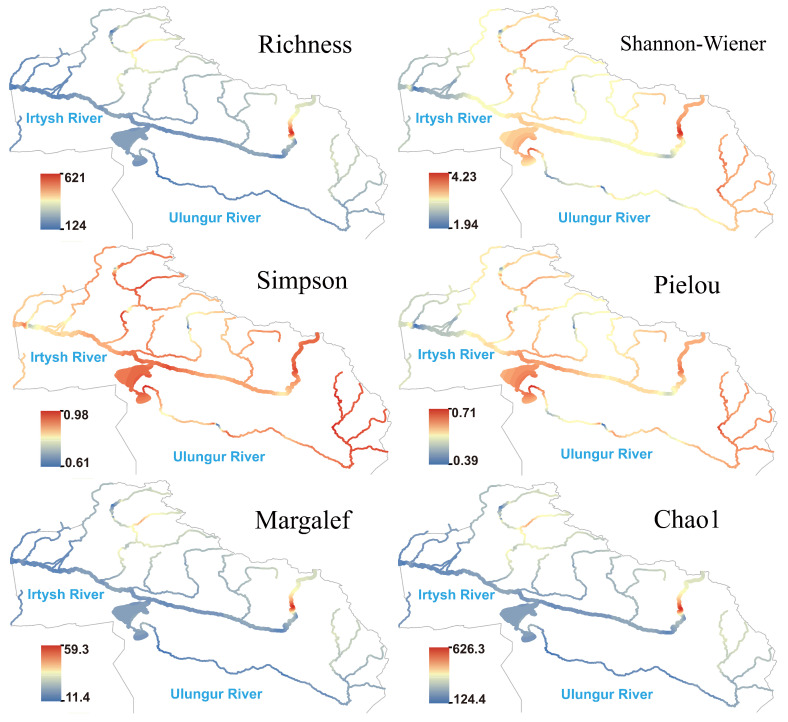
Species richness and α-diversity index of heterotrophic microorganism in the Irtysh River basin.

**Figure 8:**
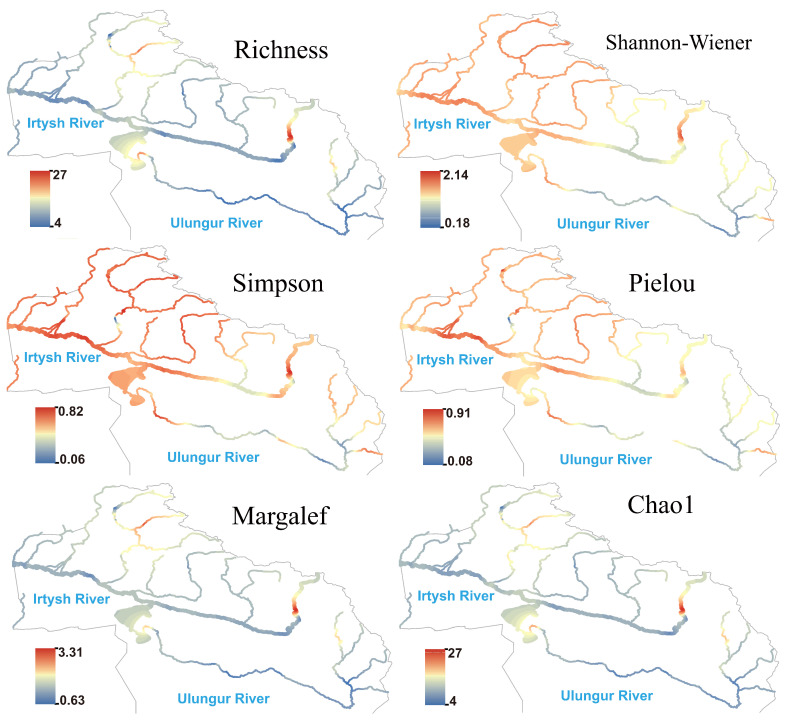
Species richness and α-diversity index of autoBacteria in the Irtysh River basin.

The spatial pattern of heterotrophic microorganism differed significantly from the above three groups of eukaryotic plankton: species richness, Margalef, and Chao1 indices were generally low in the middle and lower reaches of the main stream of the Irtysh River (Figure 7), showing a large-scale low-hotspot area; significant hotspots formed in the upper reaches of the eastern section, suggesting potentially higher organic matter supply in this reach. The Simpson index maintained moderate to high values in most reaches; the Shannon and Pielou indices slightly rebounded in the middle reaches of the main stream of the Irtysh River and were relatively high in the upper reaches of both the Irtysh River and Ulungur River. Hotspot areas of species richness, Chao1, and Margalef indices for autotrophic microorganism were consistent with those of heterotrophic microorganism (Figure 8). The Shannon, Simpson, and Pielou indices showed higher values in the Irtysh River and lower values in the Ulungur River.

The sampling sites were divided into communities with non-native fish and communities without non-native fish according to whether non-native fish were recorded and six α-diversity indices (species richness, Shannon–Wiener, Simpson, Pielou, Margalef, Chao1) were compared for algae, protozoan, fungi, heterotrophic microorganisms, and autotrophic microorganisms. Differences between groups were evaluated using the Kruskal–Wallis test (Figure S2).

In the presence of non-native fish, algae showed significantly increased indices related to species number: species richness, Margalef index, and Chao1 index were all significantly higher than those in communities without non-native fish; indices related to evenness were not significant. This indicates that the distribution of non-native fish is associated with the expansion of the number of algal species but has a limited impact on the uniformity of the community.

Contrary to algae, protozoan showed a significant decrease in species number in the presence of non-native fish: species richness, Margalef, and Chao1 indices were significantly reduced; the Simpson index also weakened, while differences in Shannon and Pielou indices were not significant. Fungi consistently showed lower species numbers in communities with non-native fish, with significantly reduced species richness, Margalef, and Chao1 indices, while there were no significant differences in Shannon, Simpson, and Pielou indices. This implies that the distribution of non-native fish is associated with a “decline in the richness dimension” of fungi, but the overall evenness has not undergone systematic changes.

The presence of non-native fish was associated with a general reduction in the α-diversity of heterotrophic microorganisms: species richness, Margalef, and Chao1 were all significantly reduced, and the Shannon and Simpson indices also significantly decreased, with no significant difference in the Pielou index. This result indicates that heterotrophic microorganisms generally showed a pattern of low abundance and uneven distribution in reaches with non-native fish. Autotrophic microorganisms showed no significant differences in indices related to species number (species richness, Margalef, Chao1 indices), but slight changes occurred in diversity/evenness indices: the Shannon index significantly decreased, while the Simpson index significantly increased, with no significant difference in the Pielou index. This suggests that the species number of autotrophic microorganisms remained basically unchanged in reaches with non-native fish, but the dominant structure underwent subtle adjustments.

### 3.3. Contributions of Natural and Anthropogenic Factors to Diversity

The results of the random forest model showed that the interpretability of the α-diversity indices varied between different groups of eukaryotic plankton and prokaryotic microorganisms. For species richness and the Margalef index, the model exhibited higher explanatory power, with an overall range of 29–56%. In contrast, the explanatory power for evenness-related indices (Shannon, Simpson, Pielou) was relatively lower. This indicates that environmental and biological factors play a more significant role in driving community patterns at the level of species number, while their ability to explain community evenness patterns is limited.

**Figure 9:**
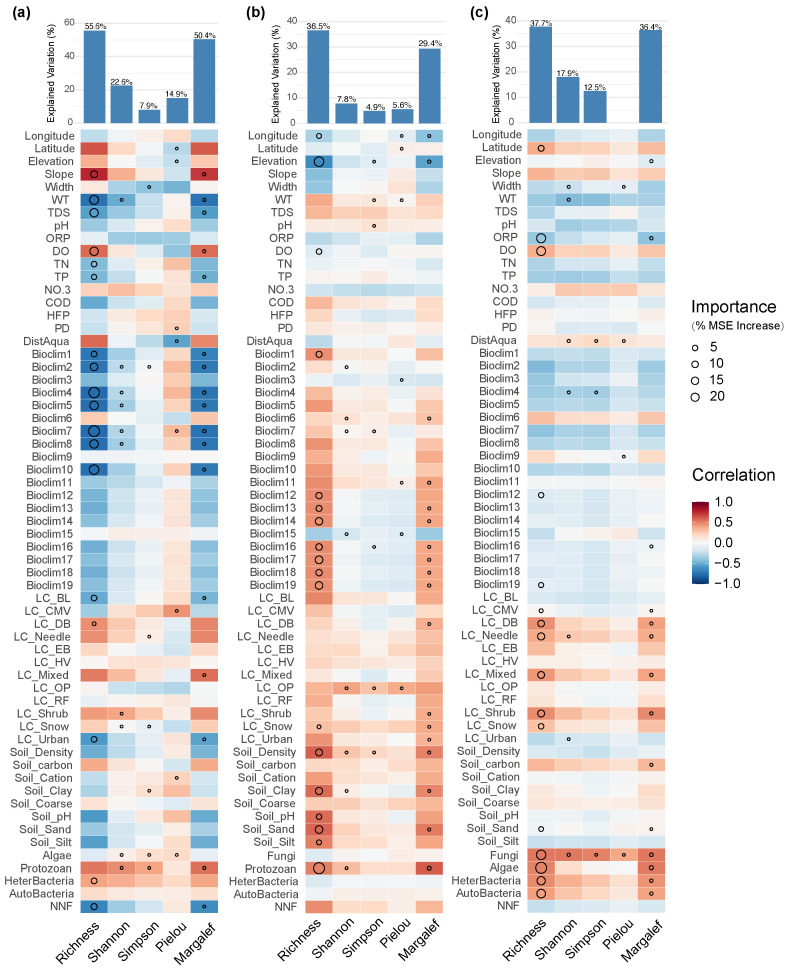
Major factors determining (**a**) fungi; (**b**) algal; (**c**) protozoan α-diversity in the Irtysh River basin.

The diversity of fungi communities was primarily driven by water physicochemical factors (e.g., water temperature, total dissolved solids, dissolved oxygen, total phosphorus) and temperature-related climatic factors (Bioclim1–10) (Figure 9a). However, unlike the aforementioned groups, non-native fish richness showed a significant negative effect on fungi species richness and the Margalef index. This suggests that non-native fish may directly reduce fungi species pools and the maintenance of rare taxa through mechanisms such as sediment disturbance and alteration in organic matter cycling.

The diversity of the algal community was mainly driven by geographic location (longitude, altitude), the richness of protozoans, and the bioclimatic factors related to precipitation (Bioclim12–19) (Figure 9b). Additionally, some soil properties (e.g., fine-soil bulk density, clay and sand content) exerted significant effects on algal communities. Among all variables, altitude and protozoan richness were the most important, indicating that algal diversity was largely shaped by the combined effects of geographical gradients and food web relationships. Notably, non-native fish richness did not emerge as an important factor for any algal or protozoan indices, suggesting that its direct effects on these two groups were limited, and it was more likely to influence community patterns indirectly by altering food web structure and competitive relationships.

Among eukaryotic plankton, protozoan-community diversity was primarily influenced by the richness of other biological groups and land-use types (Figure 9c). In the model, cover types such as mixed forests, shrubs, coniferous forests, and deciduous broad-leaved forests all showed high importance for protozoan richness and the Margalef index. This indicates that protozoan communities are particularly sensitive to biological interactions and terrestrial landscape patterns.

**Figure 10:**
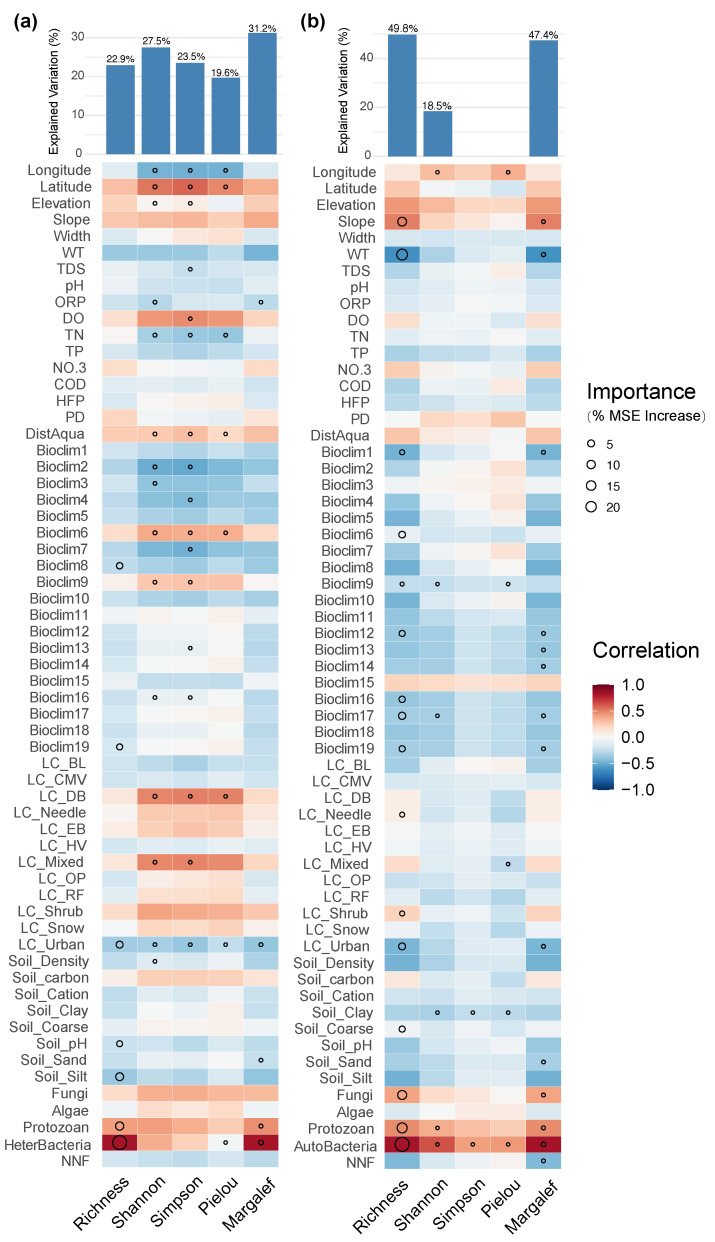
Major factors determining (**a**) autotrophic microorganism and (**b**) heterotrophic microorganism α-diversity in the Irtysh River basin.

For prokaryotic microorganism, the richness and Margalef index of both autotrophic and heterotrophic communities were significantly affected by urbanization level, the richness of other biological groups, and protozoan diversity (Figure 10). In addition, water temperature and some bioclimatic factors related to precipitation also acted as important drivers in the model of heterotrophic microorganisms. Autotrophic microorganisms, in terms of the Shannon and Simpson indices, were mainly influenced by geographical location (latitude and longitude), water physicochemical properties (dissolved oxygen, total nitrogen), and distance from aquaculture sites, suggesting that improved nutrient levels and dissolved oxygen conditions may have facilitated the dispersal and coexistence of autotrophic microorganism communities.

### 3.4. Impacts of Key Environmental Factors on Diversity

Further linear mixed-effects model (LMM) analysis validated the above research findings, which include that the driving effect of environmental and biological factors on community species richness was significantly higher than that on community evenness, and that invasive fish could inhibit the richness of fungi and protists. In addition, the model analysis also revealed that different taxa responded differently to environmental and biological factors: specifically, fungal communities were more sensitive to water physicochemical factors such as water temperature and dissolved oxygen, as well as temperature-related climatic factors, while algal communities respond more strongly to geographical location and precipitation-related factors. Overall, biological factors have the most prominent explanatory power for community species richness, and there are extensive positive and negative coupling relationships among different communities; at the same time, water physicochemical conditions, soil properties, and the intensity of human activities also have non-negligible impacts on community structure.

**Figure 11:**
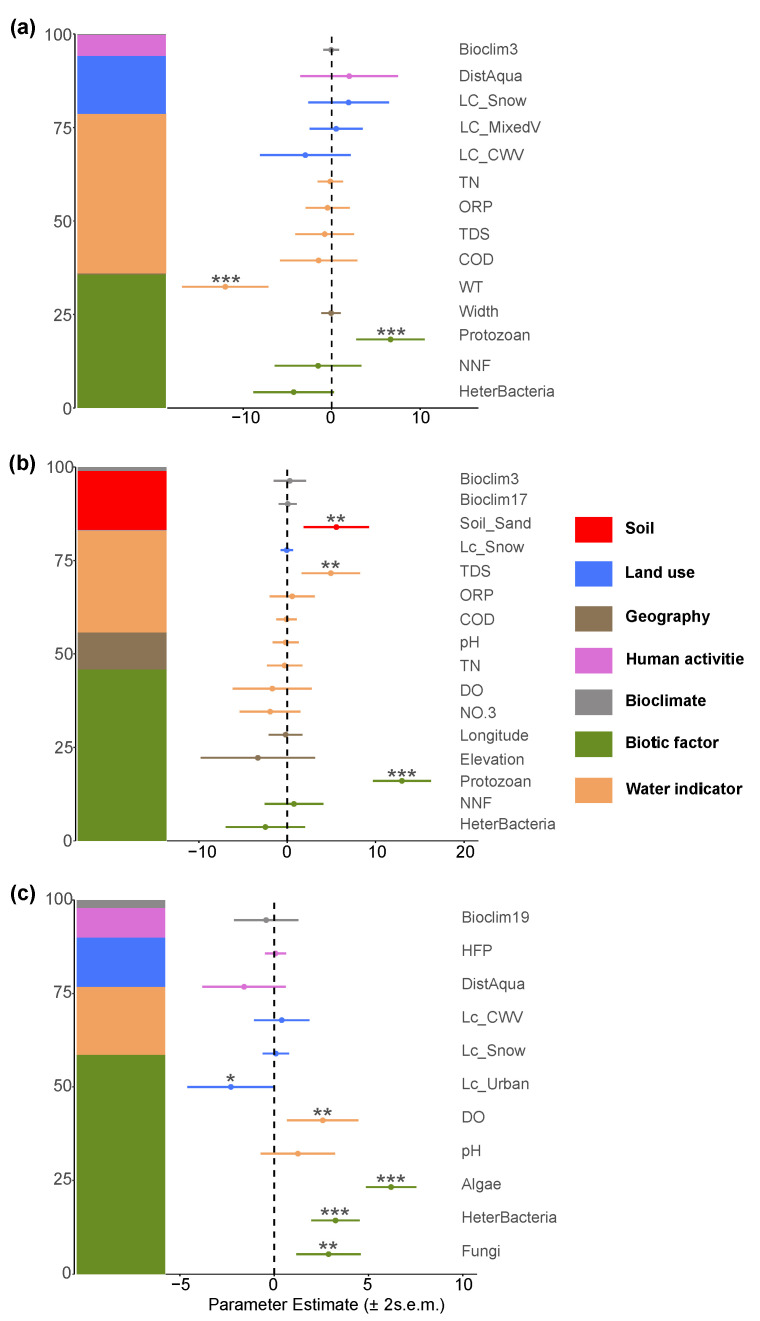
Major factors determining (**a**) fungi; (**b**) algal; (**c**) protozoan richness in the Irtysh River basin. The symbols *, **, and *** indicate statistical significance at the levels of p<0.05, p<0.01, and p<0.001, respectively.

In the fungi community, the negative effect of non-native fish was revalidated, further confirming their significant inhibitory role on fungi diversity, which is consistent with the results of the random forest model (Figure 11a). Concurrently, fungi richness showed a positive correlation with protozoa, suggesting a potential close ecological coupling between the two groups.

In the algal community, the effect of non-native fish differed from that on fungi, showing a positive relationship. Additionally, algal richness exhibited a strong positive correlation with protozoa, while a significant negative correlation was observed with heterotrophic microorganisms (Figure 11b). Total dissolved solids in water also had a positive effect on the number of algal species, further emphasizing the important regulatory role of water quality conditions on algal communities.

The richness of the protozoan community was primarily influenced positively by other biological taxa, particularly by its relationships with algae, fungi, and heterotrophic microorganisms, indicating a high degree of interactivity among biological communities. Meanwhile, the distance from aquaculture sites had a negative effect on protozoan richness, suggesting that human aquaculture activities may potentially disrupt protozoan-community diversity (Figure 11c). No significant effect of non-native fish was detected in this group.

**Figure 12:**
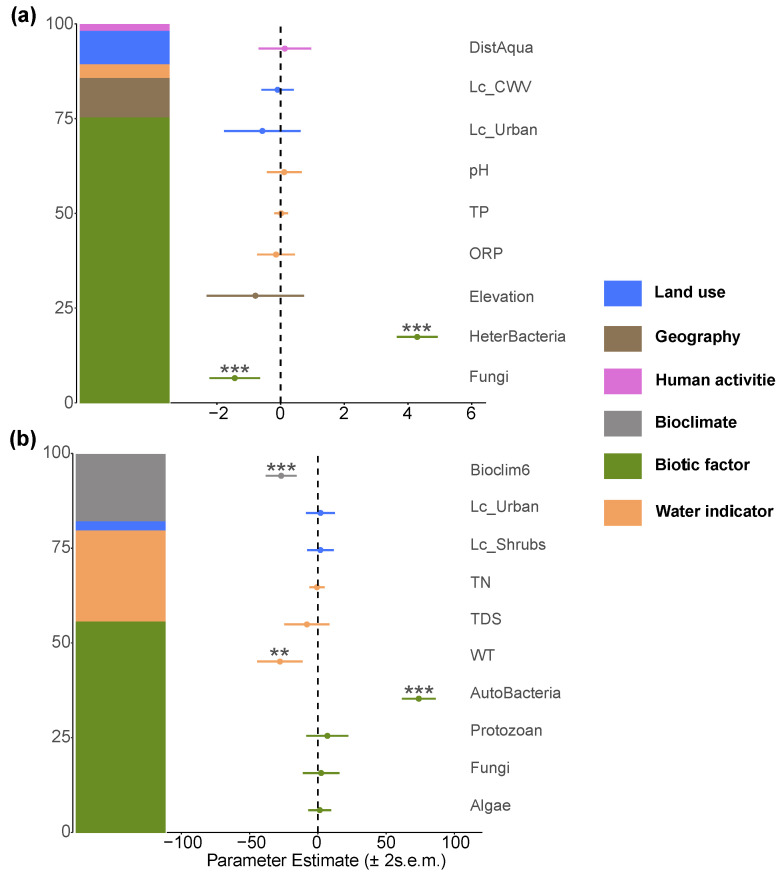
Major factors determining (**a**) autotrophic microorganism and (**b**) heterotrophic microorganism richness in the Irtysh River basin. The symbols **, and *** indicate statistical significance at the levels of p<0.01, and p<0.001, respectively.

In the autotrophic microorganism community, the diversity of heterotrophic microorganisms showed a strong positive correlation with it, while fungi exerted a negative effect. The positive impact of population density on autotrophic microorganism richness suggests that human activities may indirectly promote their diversity through nutrient input. The richness of the heterotrophic microorganism community was driven by a strong positive coupling effect with autotrophic microorganisms, and fungi also showed a significant positive effect. However, population density had a negative impact on heterotrophic microorganisms, indicating that increased intensity of human activities may inhibit their community structure. Notably, non-native fish did not show significant effects on either autotrophic or heterotrophic microorganism communities (Figure 12).

Although the richness of exotic fish was considered in the model averaging for the optimal models of fungi and algae, it did not have a significant effect. This suggests that, among biotic factors, the richness of other planktonic groups better explains species richness at similar trophic levels. The richness of exotic fish showed a positive correlation with algal diversity indices; however, it had no significant direct effect on protozoan or prokaryotic microbial communities and was therefore excluded from the set of optimal models. Collectively, these results indicate that the effects of exotic fish on planktonic microbial diversity are mechanistically diverse, and the pathways through which they influence different groups may vary considerably.

### 3.5. Impacts of Natural and Anthropogenic Factors on Communities

#### 3.5.1. Distance Decay Patterns of Basin Biological Communities

Results of the Mantel test showed that community dissimilarities of different taxa in the basin were significantly correlated with both geographical distance and environmental distance, but the strength and significance varied with taxa and the presence or absence of non-native fish. Overall, environmental distance generally exhibited stronger explanatory power for community composition than geographical distance. Among all taxa, the algal community had the highest correlation with environmental distance, followed by fungi, fish, heterotrophic microorganisms, and autotrophic microorganisms. Protozoans were an exception, as they showed a stronger overall response to geographical distance and a relatively weaker response to environmental distance (Figure S3).

When comparing communities with and without non-native fish, the distance decay patterns of different taxa showed distinct characteristics. For autotrophic microorganisms and heterotrophic microorganisms, the presence of non-native fish enhanced their correlation with environmental distance, indicating that the introduction of non-native fish may have indirectly affected the structure of the microbial community by altering the environmental conditions of the water (Figure S3). In contrast, for protozoan and fungi communities, the correlations with geographical and environmental distances were non-significant in the presence of non-native fish, whereas both showed significant and strong correlations with geographical and environmental distances in the absence of non-native fish. This suggests that non-native fish weakened the original spatial structure of fungi and protozoan communities. For algae, the correlation with environmental distance remained high regardless of the presence of non-native fish; without non-native: Mantel, indicating that algal communities are mainly driven by environmental conditions. The distance–decay relationship of the fish community itself was relatively weak and further weakened in the presence of non-native fish; environment: Mantel, suggesting that non-native fish may have reshaped the spatial pattern of native fish communities.

In summary, the distance decay patterns of basin communities exhibit significant taxonomic differences: algal communities are most dependent on environmental dissimilarities; the environmental dependence of microbial communities is significantly enhanced in the presence of non-native fish; and the spatial structure of fungi and protozoan communities is weakened under non-native fish invasion.

#### 3.5.2. Stressor Affecting Basin Biological Communities

**Figure 13:**
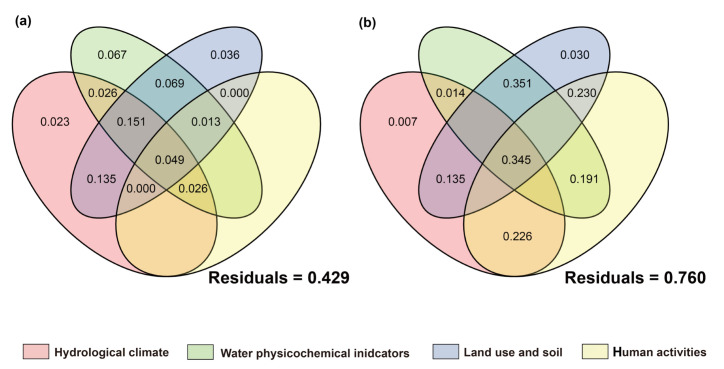
Proportion of contributions of natural and anthropogenic factors to fish communities as quantified by variance partitioning analysis. VPA under the condition of (**a**): introduced-fish community; (**b**): native-fish community.

**Figure 14:**
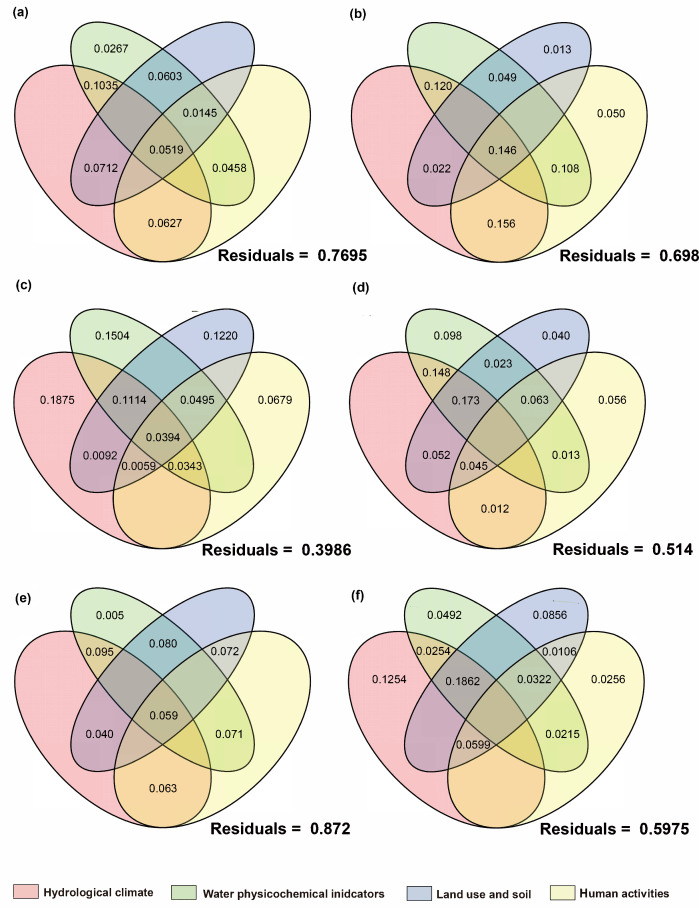
Proportion of contributions of natural and anthropogenic factors to eukaryotic plankton communities as quantified by variance partitioning analysis. The right side represents the introduced-fish community, and the left side represents the native-fish community. (**a**,**b**): Algae; (**c**,**d**): fungi; (**e**,**f**): protozoan.

**Figure 15:**
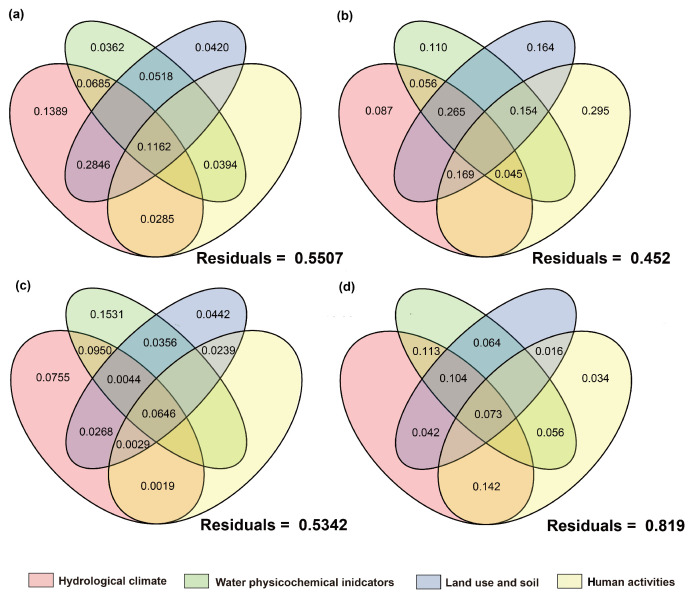
Proportion of contributions of natural and anthropogenic factors to prokaryotic microbial communities as quantified by variance partitioning analysis. The right side represents the introduced-fish community, and the left side represents the native-fish community. (**a**,**b**): Autotrophic microorganisms; (**c**,**d**): heterotrophic microorganisms.

The results of variance partitioning analysis (VPA) showed that hydrological climate, water physicochemical parameters, land use and soil, human activities, and other factors collectively explained the variations in community composition, but the relative influences of natural and anthropogenic factors differed among different taxa (Figure 13, Figure 14 and Figure 15). Regardless of the introduction of non-native fish, all taxa exhibited a high proportion of unexplained variation (39.9–87.2%), indicating that in addition to environmental and human activity factors, historical processes, interspecific interactions, and stochastic processes are also important regulatory factors.

For communities with non-native fish, the single-factor contribution of human activities was generally absent. Algae showed high responses to environmental factors with a relatively low unexplained proportion (39.9%). The explained variation was mainly attributed to the individual effects of hydrological climate, water physicochemical properties, and land use factors, while the contributions of human activity factors and their interactions were relatively low. This phenomenon was also observed in prokaryotic microorganisms. In contrast, the net effects of hydrological climate, land use, and human activity factors on fungi and protozoan communities were negative, with the main explanations derived from interactions between factors. The high unexplained proportions (fungi: 76.9%; protozoa: 87.2%) suggested that they were more likely to be influenced by unmeasured factors or intracommunity ecological processes.

In communities without non-native fish, the single-factor contribution of human activities and their related multi-factor interactions were generally present. The net effects of hydrological climate, land use, and physicochemical parameters on heterotrophic microbial communities were all negative, with a high residual (81.9%), indicating that the existing environmental and anthropogenic factors had relatively limited explanatory power for their communities. Fungi, which are also decomposers, follow the same pattern, with their main explanations also derived from interactions between factors. This indicated that the variation in fungi communities is difficult to be driven by a single factor but is the result of the combined action of multiple environmental gradients. The fish community with non-native species had a relatively low unexplained proportion (42.9%), mainly contributed by land use-related multi-factor interactions. Human activities and land-use factors played a certain role in explaining the composition of fish communities without non-native species, which is consistent with the ecological characteristic that fish are sensitive to anthropogenic disturbances.

In summary, although each biological community is jointly shaped by environmental and anthropogenic gradients, the explanatory power of a single factor is limited and the explanatory proportion of human activity factors is relatively weak. Algal communities are more sensitive to environmental and anthropogenic factors, with relatively high explanatory power. In the presence of non-native fish, the explainability of fungi and protozoan communities is low; in the absence of non-native species, the explainability of fish and heterotrophic microbial communities is low. These results are also consistent with the distance–decay pattern revealed by Mantel tests: algal communities are mainly constrained by environmental factors and fish communities are more sensitive to anthropogenic disturbances, while the drivers of the community structure of microbes, fungi, and protozoa are more complex.

## 4. Discussion

This study used high-throughput sequencing technology to comprehensively map the community composition and diversity patterns of fish species and various planktonic organisms within the research area. The findings revealed distinct regularities and variations in the distribution of biodiversity indices across different biological groups.

### 4.1. Community Composition and Diversity Distribution Patterns

Regarding the fish community, the 23 confirmed species after rigorous data processing belong to six orders and 10 families, including nine non-native fish. Consistent with the environmental-DNA monitoring results of the Ulungur River basin [64], which detected invasive fish species such as *Oreochromis niloticus* and *Oreochromis mossambicus*, the water body retains characteristics of native species while being influenced to some extent by non-native species. *Phoxinus ujmonensis*, *Gobio acutipinnatus*, *Cottus sibiricus*, and *Leuciscus baicalensis* are dominant species with high sequence counts, likely due to their strong adaptability to the local aquatic environment, particularly in terms of food resource acquisition and meeting reproductive requirements. The presence of non-indigenous fish such as *Misgurnus anguillicaudatus*, *Carassius auratus*, and *Cyprinus carpio*, which may have entered the water body through human introduction or natural dispersal, can alter the competitive dynamics between native fish and potentially exert profound impacts on material cycling and energy flow throughout the ecosystem via food webs, which warrant further investigation [65].

The community compositions of eukaryotic plankton and prokaryotic microorganism exhibit rich diversity and distinct dominant group characteristics. Ciliophora dominate the protozoan community, consistent with their ecological role as important consumers in freshwater microbial food webs, participating in material cycling and energy flow [66]. The presence of predatory ciliates and mixotrophic or heterotrophic euglenoids further confirms the complexity of aquatic microbial food webs. Their interactions, including predation, competition, and other relationships, collectively maintain ecosystem stability and dynamically adjust in response to environmental changes [67].

The algal community is dominated by chlorophyta, exhibiting typical characteristics of freshwater photosynthetic plankton communities. This is closely related to the strong adaptability of chlorophytes to light and nutrient conditions in freshwater environments. As primary producers, chlorophytes provide substantial organic matter to the entire ecosystem through photosynthesis, and their productivity directly influences the survival and reproduction of subsequent trophic-level consumers [68].

Ascomycota account for a high proportion of the fungi community, predominantly saprotrophic species, indicating their key role in the degradation of organic matter in aquatic environments. They decompose organic substances such as plant and animal residues into inorganic nutrients, facilitating the cycle of material in the ecosystem. Their decomposition efficiency is influenced by environmental factors including water temperature and pH [64,69].

Cyanobacteria, as important photosynthetic autotrophs, play important roles in carbon fixation processes. The carbon they fix is transferred through the food chains, supporting the carbon cycle of the entire ecosystem [70]. Proteobacteria account for nearly half of heterotrophic prokaryotes, predominantly Gram-negative bacteria involved in organic-matter degradation, corresponding to the large amount of organic matter requiring decomposition in the water body. Their presence ensures timely degradation of organic substances and nutrient regeneration, with different heterotrophic prokaryotes potentially specializing in degrading specific organic-matter types [71].

The α-diversity indices of fish indicate significantly higher species richness in the downstream reaches compared with the middle reaches, possibly due to the larger water area, more complex habitats, and richer food resources in the downstream region. These factors provide more living and reproductive space for fish, thereby increasing species richness [72]. Environmental conditions in the middle and upper reaches, such as water-flow velocity and temperature range, may be more similar, resulting in no significant differences in other diversity indices besides species richness and maintaining relative stability in fish-community structure and composition.

Algae form stable diversity hotspots in the middle and lower reaches of the main streams of the Irtysh and Ulungur Rivers, with diversity gradually decreasing upstream. This pattern may be attributed to the greater availability of nutrients and relatively gentle water flow in the middle and lower reaches, which favor algal growth and reproduction [73]. The observed hotspots in the Shannon and Pielou indices likely result from stable environmental conditions in these regions, promoting balanced interspecific competition and reducing the dominance of any single species.

Protozoan diversity hotspots extend further into northern high-altitude areas, suggesting that protozoa are more sensitive to local temperature or human activities. Lower temperatures and reduced human disturbances in high-altitude regions may provide more suitable habitats for protozoa. In contrast, most sections of the Ulungur River experience greater human impact and other environmental pressures, resulting in lower protozoan diversity. These differences reflect variations in ecological conservation and disturbance levels across different river basins.

At the river network scale, fungi exhibit higher diversity in the Irtysh River and lower diversity in the Ulungur River, potentially due to differences in vegetation cover and organic-matter input in these regions [64]. Areas with abundant vegetation provide more organic matter, which serves as a resource for fungi, thereby promoting their growth and reproduction [74]. Although some main stream sections have lower species richness, community evenness remains high. This suggests that environmental conditions in these areas may facilitate balanced coexistence among species, maintaining community stability through efficient resource allocation despite reduced species numbers.

The spatial distribution of heterotrophic microorganisms differs significantly from that of eukaryotic plankton, possibly due to a higher supply of organic matter in the upper reaches of the eastern section. An ample supply of organic matter provides abundant nutrients for heterotrophic microorganisms, promoting increased diversity. In contrast, environmental factors such as water flow and dissolved oxygen in the middle and lower reaches may contribute to lower heterotrophic microorganism diversity. Faster water flow can hinder bacterial attachment and reproduction, while variations in dissolved-oxygen levels can affect the survival of different bacterial species [71,73]. Autotrophic microorganisms exhibit higher Shannon, Simpson, and Pielou indices in the Irtysh River compared with the Ulungur River, potentially due to differences in environmental conditions that influence autotrophic microorganism growth, such as light availability and nutrient concentrations. The Irtysh River may provide more favorable conditions regarding light duration, intensity, and nutrient levels, supporting autotrophic microorganism growth and diversification [75].

The presence of non-indigenous fish exerts varying impacts on the α-diversity of different organisms, potentially due to competition or predation between non-indigenous fish and native organisms for food resources and habitat [64]. The significant increase in algal species richness indices caused by non-indigenous fish may result from altered survival pressure on algae or changed abundances of other herbivorous organisms due to predation by non-indigenous fish, indirectly promoting algal growth and species expansion [76]. Conversely, the significant decrease in species-richness indices of protozoa, fungi, and heterotrophic microorganisms may be due to direct or indirect inhibition of their growth and reproduction by non-indigenous fish, such as altered chemical environments from fish excreta that are unfavorable to these organisms [77]. Autotrophic microorganisms show stable species counts but adjusted dominant structures, indicating different response strategies compared with other groups, potentially adapting to influences from non-indigenous fish through changes in dominant species. This adaptive adjustment helps maintain functional stability of autotrophic microorganism communities [71,77].

### 4.2. The Explanation of Climatic and Environmental Factors and Biological Factors on the α-Diversity of Plankton

This study reveals the formation mechanisms of planktonic α-diversity in the Irtysh and Ulungur River basins, emphasizing the distinct roles of environmental filtering and biological interactions across different taxa. Overall, environmental factors play a more significant role in explaining species richness-related indices (species richness, Margalef index, Chao1 index), whereas variations in community evenness are more likely influenced by local disturbances and biological interaction processes. These findings align with existing studies that highlight the core regulatory roles of climate, nutrient, and hydrological conditions in shaping planktonic community patterns [78,79,80]. Additionally, climatic and environmental factors are found to be central in structuring river planktonic communities. Environmental filtering may operate through multiple pathways. For instance, fluctuations in dissolved oxygen directly affect the metabolic rates of algae and fungi, thereby altering their community composition. Dissolved oxygen is crucial in aquatic ecosystems, directly influencing the physiological activities of plankton. Studies have shown that high concentrations of dissolved oxygen can inhibit the growth of microalgae (such as *Chlorella vulgaris*), thereby affecting primary productivity [81]. Furthermore, reduced dissolved-oxygen levels threaten the survival of zooplankton populations [82]. The lack of a significant correlation between chemical-oxygen demand (a water physicochemical index) and the α-diversity of various planktonic taxa is the low level of organic pollution in the basin [83]. According to the *Environmental Quality Standards for Surface Water*, 81% (42/52) of the sampling sites in this study had COD values better than the standard Class I, while total phosphorus values at all sites exceeded the standard Class II.

On the other hand, temperature changes have extensive impacts on biological communities in freshwater ecosystems, including bacteria, fungi, and protozoans [84,85]. Temperature directly affects the growth, metabolic rate, and reproduction of fungi [86]. Some fungi species, such as aquatic filamentous fungi, have specific adaptations to low temperatures, being able to produce cold-active enzymes and antifreeze proteins, which enable them to effectively decompose organic matter even at lower temperatures [87]. Consistent with the results of this study, almost all climatic factors related to temperature and the temperature of the water are negatively correlated with the α-diversity of fungi. It has also been shown that although increased temperature may not significantly change the taxonomic diversity of fungi, it can reduce their functional diversity, especially affecting the function of decomposing litter [88]. Secondly, water temperature is a key factor determining the physical properties of water (such as density and viscosity) and biogeochemical processes (such as dissolved-oxygen concentration and nutrient cycling) [89]; temperature changes can also indirectly affect the physicochemical environment of rivers, thereby influencing fungi communities [90]. A survey of rivers in the Nile Delta region showed that air temperature and water temperature were the main factors affecting fungi distribution; in addition, ions such as calcium and magnesium are also associated with fungi communities, indicating that the comprehensive effect of water physicochemical properties is crucial for fungi community structure [90]. Freshwater fungi play key roles in decomposing underwater wood, degrading lignocellulose, and releasing nutrients, and are important components of ecosystem functions [91]. Therefore, the impact of global warming on the diversity and functions of freshwater plankton deserves attention.

Precipitation transports nutrients from terrestrial ecosystems into rivers via surface runoff, thereby influencing nutrient availability for algal growth [78,92,93]. For example, the type and distribution of land use within a catchment area affect the substances entering lakes through runoff and pollution sources. Excessive nutrient inputs, particularly nitrogen and phosphorus, are primary drivers of river eutrophication and algal blooms [94]. In some regions, climate-change-induced alterations in precipitation patterns can modify nitrogen fertilizer application rates, subsequently impacting riverine nitrogen levels and indirectly threatening ecosystems such as the Great Barrier Reef [95]. Elevated concentrations of nutrients like nitrogen and phosphorus can promote the proliferation of certain algal species but may inhibit the growth of some fungi and heterotrophic microorganisms, resulting in increased spatial heterogeneity within the community. A study of the Yellow River delta demonstrated that freshwater replenishment, an important ecological restoration strategy, indirectly influenced planktonic communities by altering environmental factors such as nitrate concentration and salinity [96].

Biological factors play a significant role, as evidenced by the clear coupling relationships observed among different taxa. For example, the positive correlation between algae and protozoa indicates a close connection between protozoan feeding activities and algal productivity [64]. Algae are the primary producers in aquatic ecosystems, supplying energy and organic matter through photosynthesis [97]. As heterotrophic organisms, many protozoan species feed on algae, bacteria, and fungi, establishing predator–prey relationships [98,99,100]. This energy transfer may result in similar diversity patterns between the two groups at the regional scale. Beyond predation, mutualistic symbiotic relationships also exist between algae and protozoan (e.g., *Paramecium bursaria* and symbiotic green algae of the genus Zoochlorella form symbionts [101], which enhance survival and allow the symbionts to reproduce more effectively in specific environments. In this relationship, algae provide organic matter to Paramecium through photosynthesis, while Paramecium offers a protected internal environment and carbon dioxide to the algae. Studies have shown that during co-evolution, algae and bacteria have developed various interaction modes ranging from mutualism to parasitism [102].

For prokaryotic microorganisms, the positive correlation between autotrophic and heterotrophic microorganisms reveals their complementarity in material cycling and metabolic pathways. autotrophic microorganisms convert inorganic nutrients into organic forms by fixing inorganic carbon and nitrogen, which are then utilized by heterotrophic microorganisms. Heterotrophic microorganisms decompose complex organic substances, releasing soluble organic matter and inorganic nutrients to nourish the growth of autotrophic microorganisms [103]. These findings are consistent with previous conclusions that biological factors play a prominent role in explaining the diversity patterns of river biological communities, emphasizing the importance of cross-taxon biological interactions in the impacts of community structure [79,104].

The effect of non-native fish richness on α-diversity indices shows high variability among different taxa. In fungi communities, its negative effect has been repeatedly verified, indicating that non-native fish may weaken the ability to maintain rare fungi by disturbing sediments or altering organic matter cycling. This mechanism has also been reported in other river systems; for example, non-native fish reduce the functional performance of decomposer communities by changing benthic environments [105]. For protozoan and prokaryotic microbial communities, the direct effect of non-native fish is not significant, suggesting that their influence may mainly be achieved through indirect pathways, such as changing water transparency, nutrient dynamics, or food-web structure. In most fish-farming systems, the composition of bacterial and microalgal communities may also be affected by other factors, including the use of feed, fertilizers, and antibiotics. These factors not only affect the fish gut microbiota but also influence the microbial communities in water and sediments [106], indicating that the activities of invasive fish in natural waters may also affect microbial ecology through similar mechanisms.

### 4.3. The Driving Mechanisms of β-Diversity Patterns

This study revealed the formation patterns of β-diversity in multitrophic-level communities across the basin through Mantel tests and VPA analyses. Overall, environmental distance exhibited generally higher explanatory power for community dissimilarities than geographic distance, indicating that environmental filtering is the main driver of community structure. This is consistent with the conclusion that environmental heterogeneity dominates community assembly in the Irtysh River basin’s aquatic ecosystems [64]. However, distinct taxonomic taxa showed significant differences in their response patterns to environmental gradients and exotic disturbances, reflecting variations in the impacts of community structure across trophic levels.

Among all taxa, algal communities displayed the strongest correlation with environmental distance, which remained stable regardless of the presence or absence of non-native fish. This suggests that the spatial patterns of algal communities are primarily determined by water physicochemical factors and nutrient status. As primary producers, algae are highly sensitive to water temperature, light, and nutrient levels such as total nitrogen and total phosphorus [107,108]. Previous studies have shown that phytoplankton can achieve large-scale dispersal through water flow in river systems, but their water physicochemical factors are still mainly constrained by environmental heterogeneity [109]. Therefore, within this basin, the β-diversity of algal communities primarily reflects environmental filtering and species replacement rather than geographic-isolation effects. VPA analyses further supported this, as the explained variation in algal communities was mainly contributed by hydrological climate, water physicochemical factors, and land use, with a relatively low proportion of unexplained variation. This indicates that algal communities are sensitive to natural environmental conditions and serve as important indices of basin environmental changes.

Microbial communities (including autotrophic and heterotrophic groups) were also primarily driven by environmental gradients, but their correlation with environmental distance significantly strengthened in the presence of non-native fish. This phenomenon suggests that non-native fish may indirectly affect the distribution patterns of microbial communities by altering water-nutrient cycling and organic-matter supply, thereby enhancing microbial responses to physicochemical environments [64,110]. Autotrophic microorganisms depend on light and inorganic nutrients, while heterotrophic microorganisms rely on organic matter sources. When non-native fish modify water-resource inputs through excretion, sediment disturbance, or regulation of zooplankton communities, the environmental dependence of microbial communities is further amplified, potentially making environmental filtering signals more prominent.

Combined with the results of this study, the introduction of non-native fish may exacerbate such environmental gradient effects. On the other hand, VPA showed that the explanatory power for heterotrophic microbial communities mainly came from multi-factor interactions, and the unexplained variation was higher in communities without non-native fish. This indicates that besides environmental filtering, their community structure is jointly influenced by historical processes, interspecific interactions, and stochastic processes.

The spatial structures of fungi and protozoan communities exhibited distinctly different patterns following the introduction of non-native fish: in the absence of non-native fish, both communities showed significant distance–decay effects, indicating that their spatial patterns were constrained by both environmental gradients and geographic isolation. However, in the presence of non-native fish, their correlations with both environmental and geographic distances became non-significant, suggesting that non-native fish invasion weakened their original spatial structures [64,110]. Fungi communities often depend on benthic substrates and organic-matter sources, while protozoa, as low-trophic-level consumers, rely on basal resources such as algae and bacteria. Non-native fish may disrupt the coupling between fungi/protozoan communities and environmental gradients by altering benthic environments, preying on zooplankton, or reshaping food-web structures. This result aligns with previous studies suggesting that exotic predators can reshape community spatial patterns through cascading effects [111,112]. Meanwhile, VPA results showed that fungi and protozoan communities in the presence of non-native fish had the lowest explainability, further indicating that their community structures were more susceptible to unmeasured factors, local disturbances, and stochasticity.

As a high-trophic-level group, fish communities overall showed a significant correlation with environmental distance, but the strength was lower than that of algae and microorganisms, and it was further weakened in the presence of non-native fish. This suggests that non-native fish alter native fish communities through competition, predation, or ecosystem engineering effects, partially offsetting the explanatory power of environmental gradients for fish-community dissimilarities [64,110]. The β-diversity partitioning results of fish communities showed a relatively higher proportion of nested components, indicating that local species loss played a more important role in the spatial patterns of fish communities. This is consistent with the characteristic that fish are highly sensitive to habitat fragmentation and human activities [113]. VPA results further indicated that fish communities had a higher explanatory proportion when non-native fish were present, while in the absence of non-native fish, they were mainly contributed by interactions between land-use and human-activity factors. This pattern reflects that fish, as a high-trophic-level group, have their community diversity patterns significantly affected by the dual effects of exotic-species interference and altered river connectivity due to basin-water conservancy development.

In summary, this study demonstrates that the β-diversity patterns of multitrophic-level communities in the basin are driven by multiple factors, including environmental filtering, non-native fish invasion, and human activities. Algal and microbial communities are primarily shaped by environmental gradients, and the presence of non-native fish further strengthens the environmental dependence of microbial communities. Fungi and protozoa lose their original spatial autocorrelation under non-native fish disturbance and are more susceptible to local processes and stochasticity. Fish communities show higher sensitivity to anthropogenic disturbances and species loss. These results are consistent with the comprehensive assessment of aquatic biodiversity drivers in the Irtysh–Ulungur River basin [64] and the DNA barcoding study highlighting the importance of molecular approaches in characterizing fish-community responses to environmental and anthropogenic changes [110], emphasizing the differential response mechanisms across trophic levels, indicating that low-trophic-level communities are more sensitive to environmental gradients, while high-trophic-level communities are more vulnerable to exotic species and human activities. Under the combined effects of non-native fish and human activities, energy flow, material cycling, and food-web structures are altered, leading to more complex driving mechanisms for the spatial patterns of basin communities. This provides new scientific bases for basin ecological protection and exotic-species management.

## 5. Conclusions

This study employed eDNA metabarcoding to systematically investigate fish and various plankton communities in the Irtysh River and Ulungur River basins. The results revealed that the biological community composition in these basins exhibits distinct freshwater ecological characteristics: the fish community consists of 23 species, including 9 non-native species. Among eukaryotic plankton, ciliates, green algae, and ascomycetes, as well as cyanobacteria and proteobacteria among prokaryotic microorganisms, constitute the key taxa in their respective communities. The spatial patterns of biodiversity show obvious group specificity: algae and fish form diversity hotspots in the middle and lower reaches, while protozoan hotspots extend to higher-altitude areas. The distribution of fungi is closely related to organic-matter input. Heterotrophic microorganisms widely form low hotspots in the middle and lower reaches of the main channel but form high hotspot areas in the eastern upper reaches. Autotrophic microorganisms and heterotrophic microorganisms are roughly consistent in hotspots related to species richness indices, but there are significant differences in evenness indices between the two basins. In addition, non-native fish have selective impacts on different groups: they increase the number of algal species, inhibit the richness of protozoa, fungi, and heterotrophic microorganisms, and only slightly adjust the dominant structure of autotrophic microorganisms. Environmental distance has stronger explanatory power than geographic distance for most groups. Protozoa are more affected by dispersal limitation, while for groups such as fungi and protozoa, unmeasured factors (e.g., interspecific interactions and historical processes) are more important, highlighting the complexity of the mechanisms. These results provide a scientific basis for ecological protection and management of the basins, emphasizing the need to balance the maintenance of habitat heterogeneity and the prevention and control of non-native species according to the sensitivity characteristics of different groups, so as to ensure the integrity and stability of ecosystem functions.

## Data Availability

Raw data, assembly, and annotation are stored at https://doi.org/10.57760/sciencedb.31386. The other data supporting this study’s findings are available from the corresponding authors upon reasonable request.

## Supplementary Materials

The following supporting information can be downloaded at https://www.mdpi.com/article/10.3390/biology14121661/s1, Table S1: Description and sources of explanatory variables used in statistical processes; Figure S1: Species accumulation curve; Figure S2: Species richness and α-diversity index under the conditions of introducing non-native fish species or not; Figure S3: Distance decay model of Bray–Curtis dissimilarity of communities along geographical distance and environmental distance. (a): All sampling sites; (b): sampling sites with non-native fish species; (c): sampling sites without non-native fish species.
