# Supplementary material for: Analysis of Multitrophic Biodiversity Patterns in the Irtysh River Basin Based on eDNA Metabarcoding
Source: Biology (Basel). 2025 Nov 24;14(12):1661. doi: 10.3390/biology14121661 (PMC12730370; doi:10.3390/biology14121661)
Supplement: Supplementary file 1 [file biology-14-01661-s001.zip › biology-3946897-supplementary.pdf]

Supplementary Data

Table S1 Description and sources of explanatory variables used in statistical processes				
**Category**	**Variable**	**Unit**	**Abbreviation**	**Data Sourc**
Geography	Longitude	o	Longitude	The present study
	Latitude	o	Latitude	The present study
	Elevation	**m**	Elevation	The present study
	Slope	º*100	Slope	HydroSHEDS (Lehner et al. 2008)
	Width	**m**	Width	The present study
Water quality	Water temperature	℃	WT	The present study
	Total dissolved solids	mg/L	TDS	The present study
	pH	—	pH	The present study
	Oxidation-reduction potential	mV	ORP	The present study
	Dissolved oxygen	mg/L	DO	The present study
	Total nitrogen	mg/L	TN	The present study
	Total phosphorus	mg/L	TP	The present study

	Nitrate nitrogen	mg/L	NO.3	The present study
	Chemical oxygen demand	mg/L	COD	The present study
Human activity	Human footprint value	—	HFP	Venter et al. 2016
	Population density	people/km2	PD	WorldPop
	Distance from aquaculture site	m	DistAqua	The present study
Hydroclimate	Bioclim 1-Annual Mean Upstream Temperature	°C*10	Bioclim1	WorldClim (Hijmans et al. 2005)
	Bioclim 2 - Mean Upstream Diurnal Range	°C*10	Bioclim2	WorldClim (Hijmans et al. 2005)
	Bioclim 3 - Upstream Isothermality (Bioclim 2 / Bioclim 7) (* 100)	°C*10	Bioclim3	WorldClim (Hijmans et al. 2005)
	Bioclim 4 - Upstream Temperature Seasonality (standard deviation *100)	°C*10	Bioclim4	WorldClim (Hijmans et al. 2005)
	Bioclim 5 - Maximum Upstream Temperature of Warmest Month	°C*10	Bioclim5	WorldClim (Hijmans et al. 2005)
	Bioclim 5 - Maximum Upstream Temperature of Warmest Month	°C*10	Bioclim6	WorldClim (Hijmans et al. 2005)
	Bioclim 7 - Upstream Temperature Annual Range (Bioclim 5 - Bioclim 6)	°C*10	Bioclim7	WorldClim (Hijmans et al. 2005)
	Bioclim 8 - Mean Upstream Temperature of Wettest Quarter	°C*10	Bioclim8	WorldClim (Hijmans et al. 2005)
	Bioclim 9 - Mean	°C*10	Bioclim9	WorldClim

	Upstream Temperature of Driest Quarter			(Hijmans et al. 2005)
	Bioclim 10 - Mean Upstream Temperature of Warmest Quarter	°C*10	Bioclim10	WorldClim (Hijmans et al. 2005)
	Bioclim 11 - Mean Upstream Temperature of Coldest Quarter	°C*10	Bioclim11	WorldClim (Hijmans et al. 2005)
	Bioclim 12 - Annual Upstream Precipitation	°C*10	Bioclim12	WorldClim (Hijmans et al. 2005)
	Bioclim 13 - Upstream Precipitation of Wettest Month	°C*10	Bioclim13	WorldClim (Hijmans et al. 2005)
	Bioclim 14 - Upstream Precipitation of Driest Month	°C*10	Bioclim14	WorldClim (Hijmans et al. 2005)
	Bioclim 15 - Upstream Precipitation Seasonality (Coefficient of Variation)	°C*10	Bioclim15	WorldClim (Hijmans et al. 2005)
	Bioclim 16 - Upstream Precipitation of Wettest Quarter	°C*10	Bioclim16	WorldClim (Hijmans et al. 2005)
	Bioclim 17 - Upstream Precipitation of Driest Quarter	°C*10	Bioclim17	WorldClim (Hijmans et al. 2005)
	Bioclim 18 - Upstream Precipitation of Warmest Quarter	°C*10	Bioclim18	WorldClim (Hijmans et al. 2005)
	Bioclim 19 - Upstream Precipitation of Coldest Quarter	°C*10	Bioclim19	WorldClim (Hijmans et al. 2005)
Land use	Evergreen/deciduous needleleaf trees	%	LC_Needle	Consensus Land cover (Tuanmu et al. 2014)
	Evergreen broadleaf	%	LC_EB	Consensus

	trees			Land cover (Tuanmu et al. 2014)
	Deciduous broadleaf trees	%	LC_DB	Consensus Land cover (Tuanmu et al. 2014)
	Mixed/other trees	%	LC_Mixed	Consensus Land cover (Tuanmu et al. 2014)
	Shrubs	%	LC_Shrub	Consensus Land cover (Tuanmu et al. 2014)
	Herbaceous vegetation	%	LC_HV	Consensus Land cover (Tuanmu et al. 2014)
	Cultivated and managed vegetation	%	LC_CMV	Consensus Land cover (Tuanmu et al. 2014)
	Regularly flooded shrub/herbaceous vegetation	%	LC_RF	Consensus Land cover (Tuanmu et al. 2014)
	Urban/built-up	%	LC_Urban	Consensus Land cover (Tuanmu et al. 2014)
	Snow/ice	%	LC_Snow	Consensus Land cover (Tuanmu et al. 2014)
	Barren lands/sparse vegetatio	%	LC_BL	Consensus Land cover (Tuanmu et al.

				2014)
	Open water	%	LC_OP	Consensus Land cover (Tuanmu et al. 2014)
Soil	Soil organic carbon	g/kg	Soil_carbon	Hengl et al. 2014
	Soil pH in H2O	pH*10	Soil_pH	Hengl et al. 2014
	Sand content mass fraction	%	Soil_Sand	Hengl et al. 2014
	Silt content mass fraction	%	Soil_Silt	Hengl et al. 2014
	Clay content mass fraction	%	Soil_Clay	Hengl et al. 2014
	Coarse fragments (>2 mm fraction) volumetric	%	Soil_Coarse	Hengl et al. 2014
	Cation exchange capacity	cmol/kg	Soil_Cation	Hengl et al. 2014
	Bulk density of the fine earth fraction	cmol/kg3	Soil_Density	Hengl et al. 2014
Biotic factors	Fungi diversity	—	Fungi	The present study
	Algae diversity	—	Algae	The present study
	Protozoan diversity	—	Protozoan	The present study
	Heterotrophic bacteria diversity	—	HeterBacteria	The present study
	Autotrophic bacteria diversity	—	AutoBacteria	The present study
	Non-native fish diversity	—	NNF	The present study

Figure S1

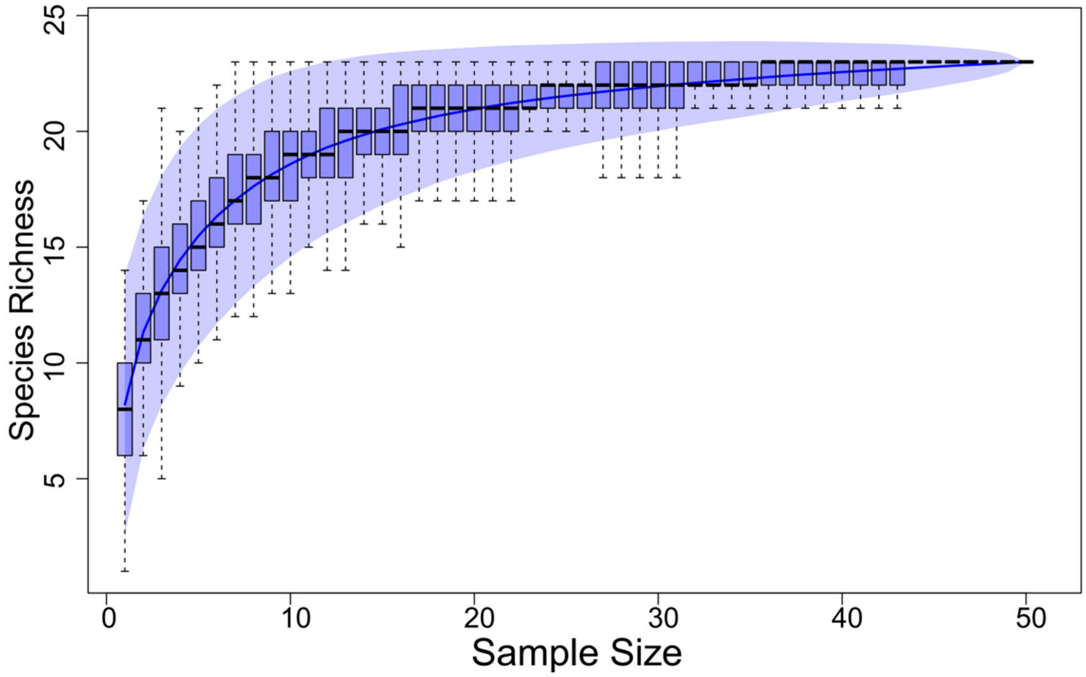

1. Accumulation curve of fish

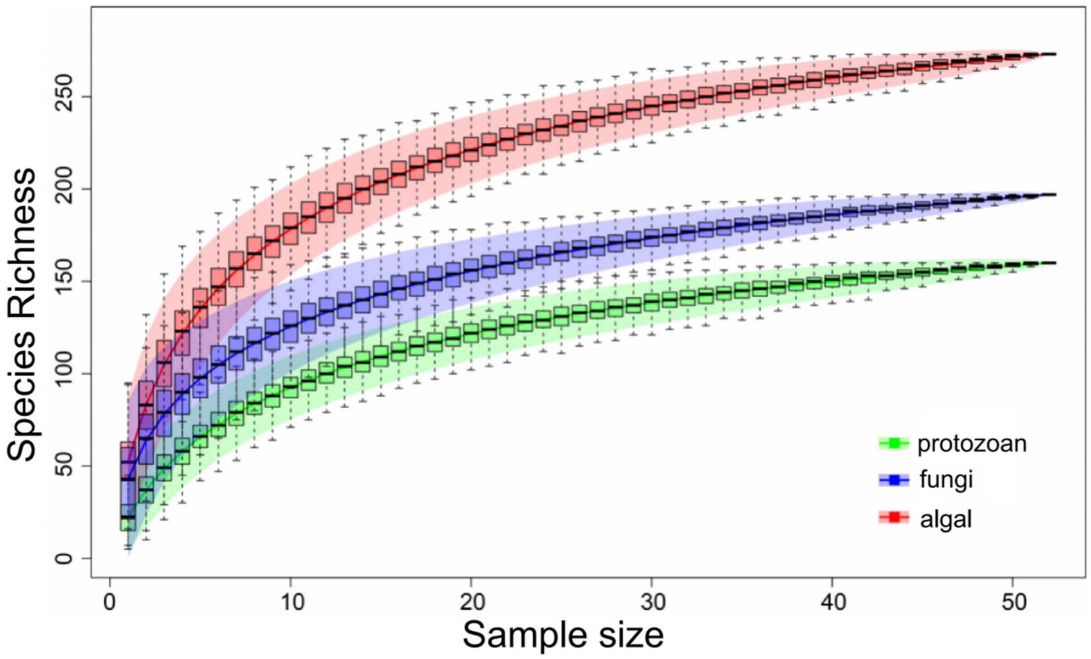

2. Accumulation curve of eukaryotic plankton

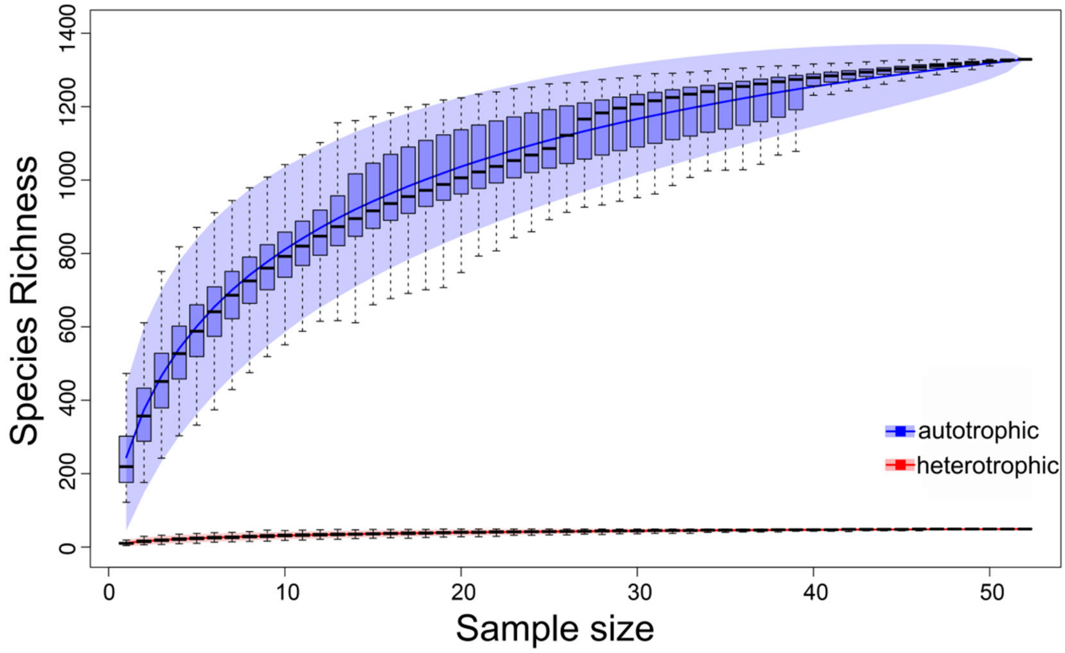

3. Accumulation curve of prokaryotic microorganisms

Figure S2

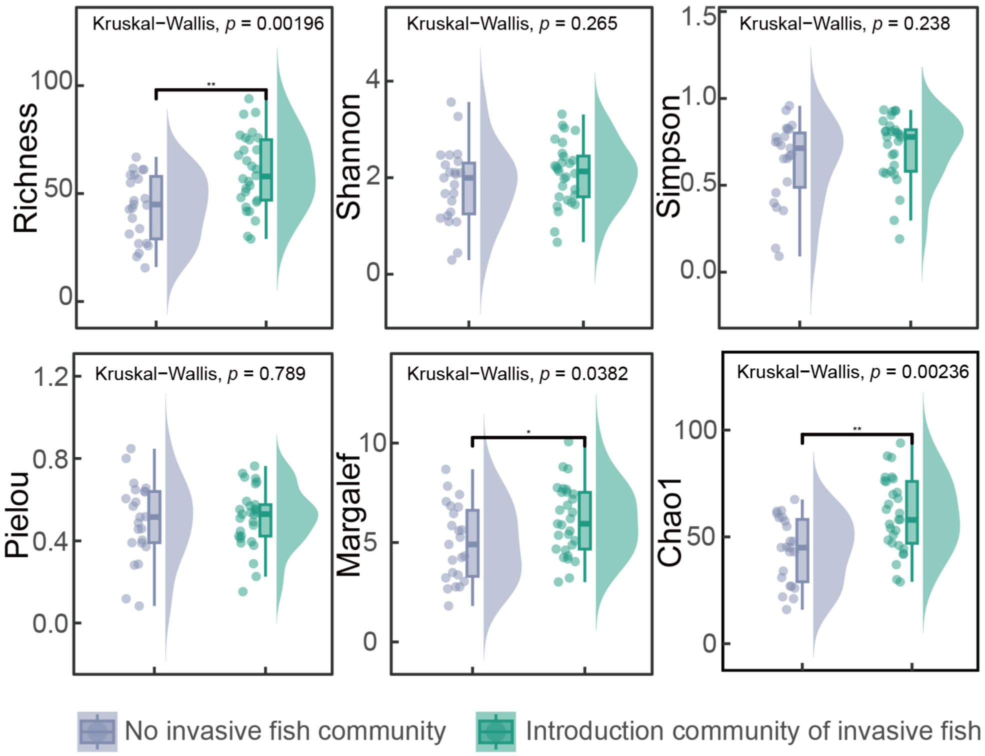

1. Species richness and α-diversity index of algae under the conditions of introducing non-native fish species or not.

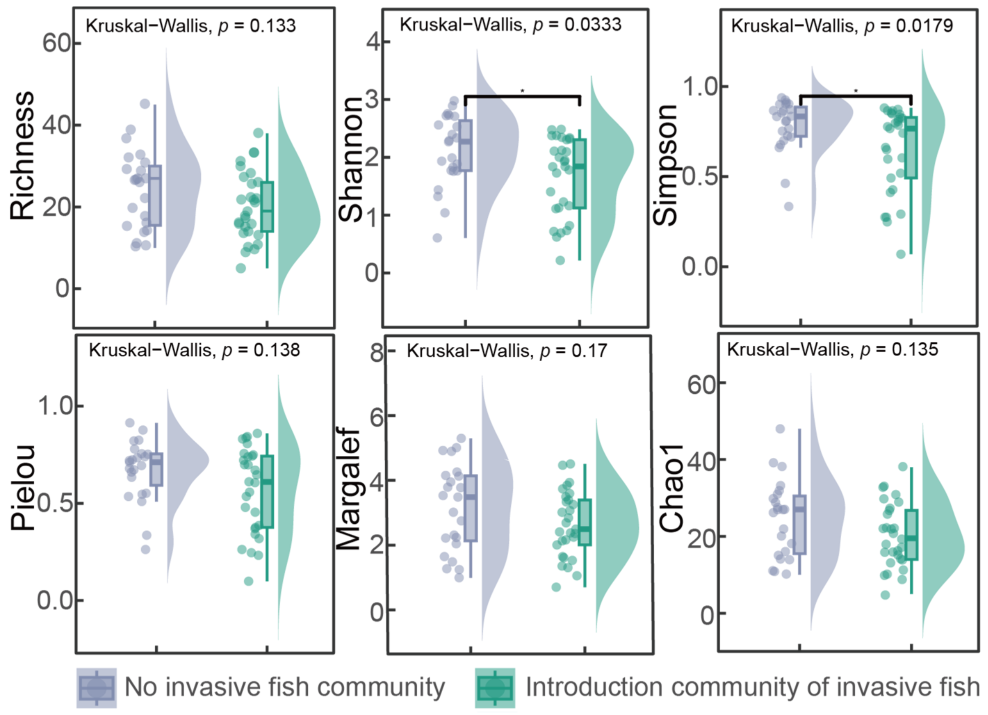

2. Species richness and α-diversity index of protozoa under the conditions of introducing non-native fish species or not.

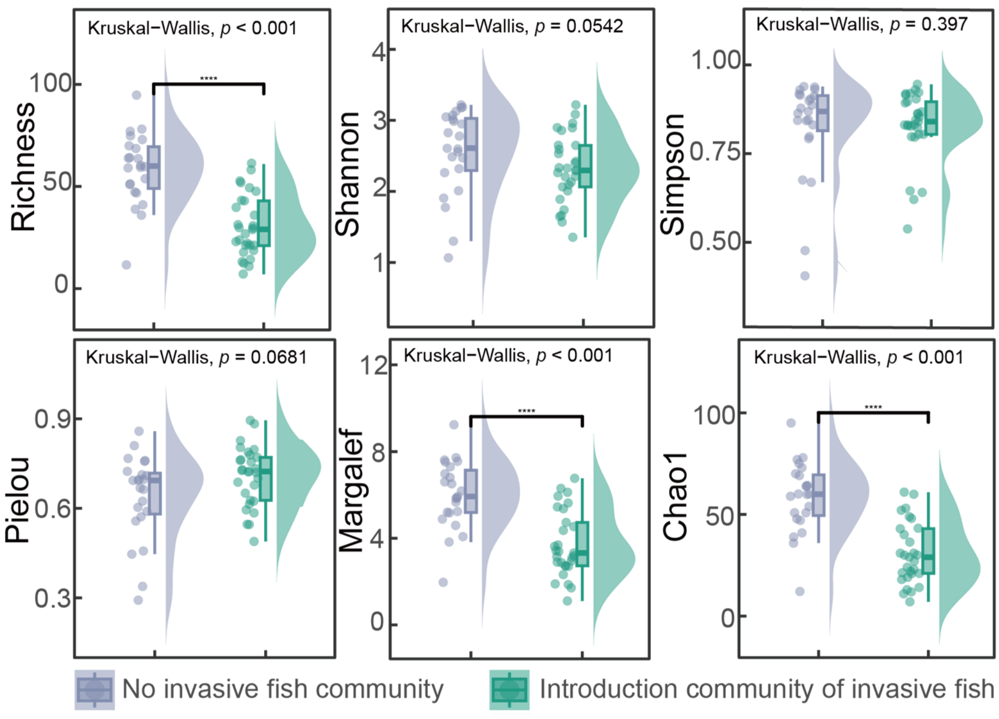

3. Species richness and α-diversity index of fungi under the conditions of introducing non-native fish species or not.

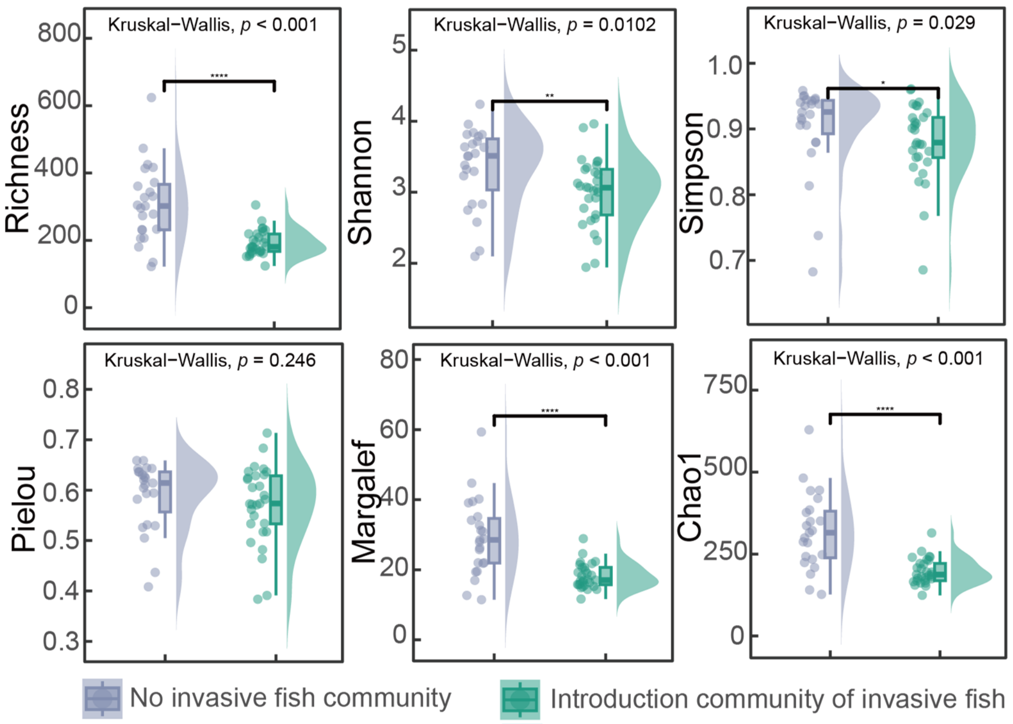

4. Species richness and α-diversity index of heterotrophic under the conditions of introducing non-native fish species or not.

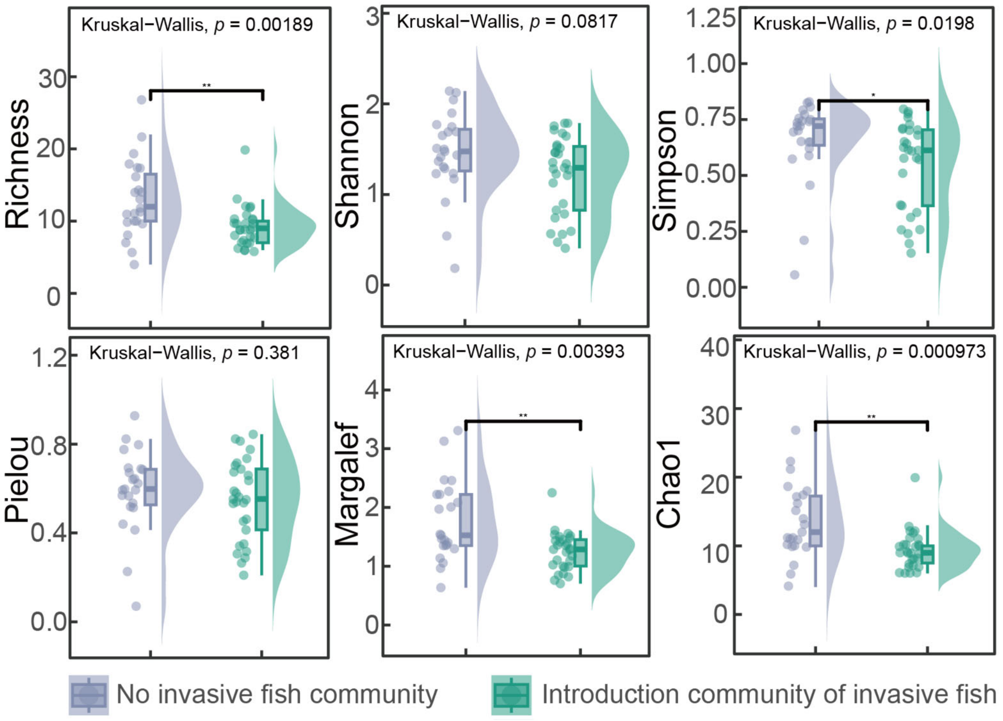

5. Species richness and α-diversity index of autotrophic under the conditions of introducing non-native fish species or not.

Figure S3

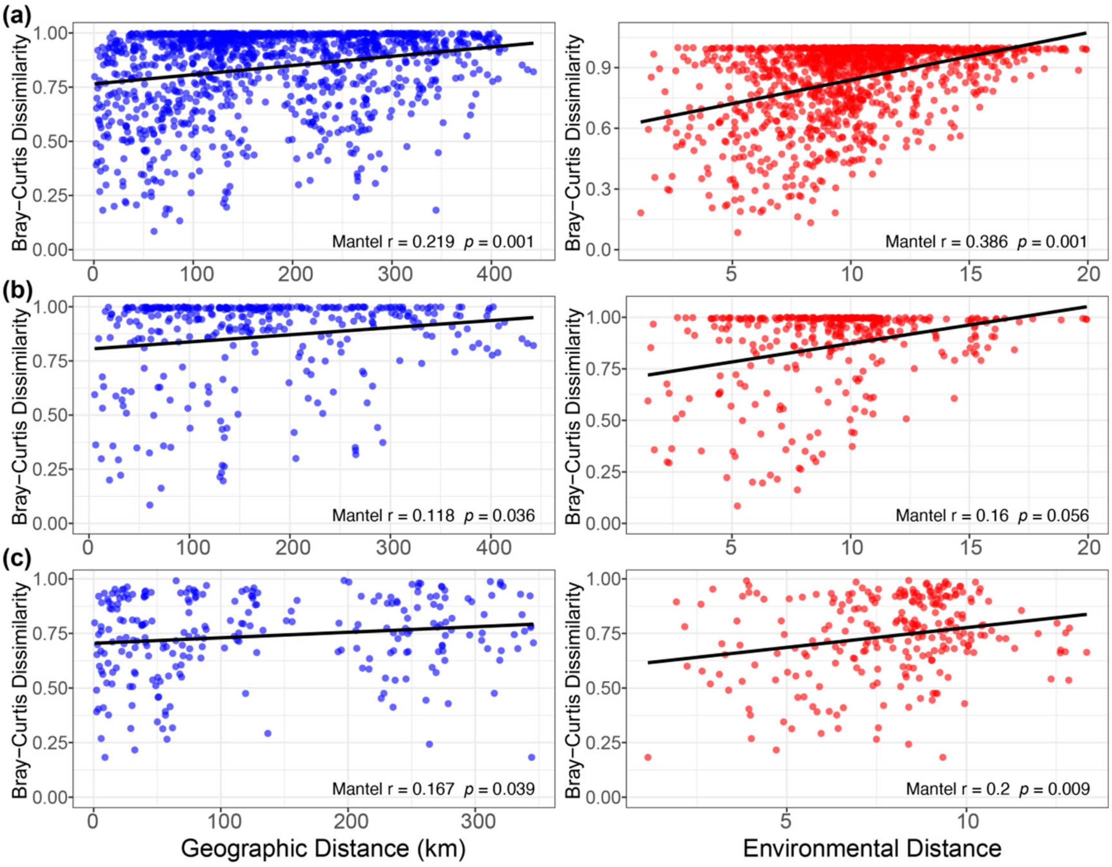

6. Distance attenuation model of Bray-Curtis dissimilarity of fish communities along geographic distance and environmental distance. (a): all sampling sites; (b): with non-native fish species; (c): with out non-native fish species.

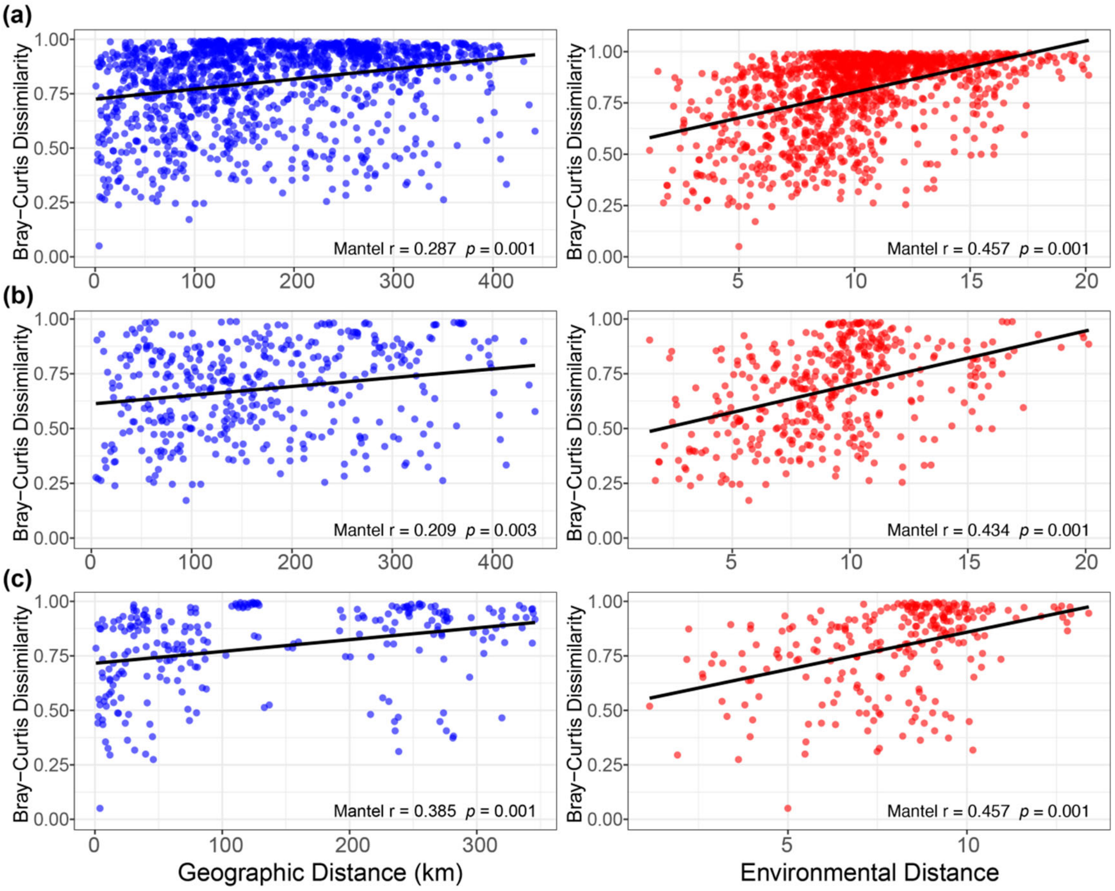

7. Distance attenuation model of Bray-Curtis dissimilarity of algal communities along geographic distance and environmental distance. (a): all sampling sites; (b): with non-native fish species; (c): with out non-native fish species.

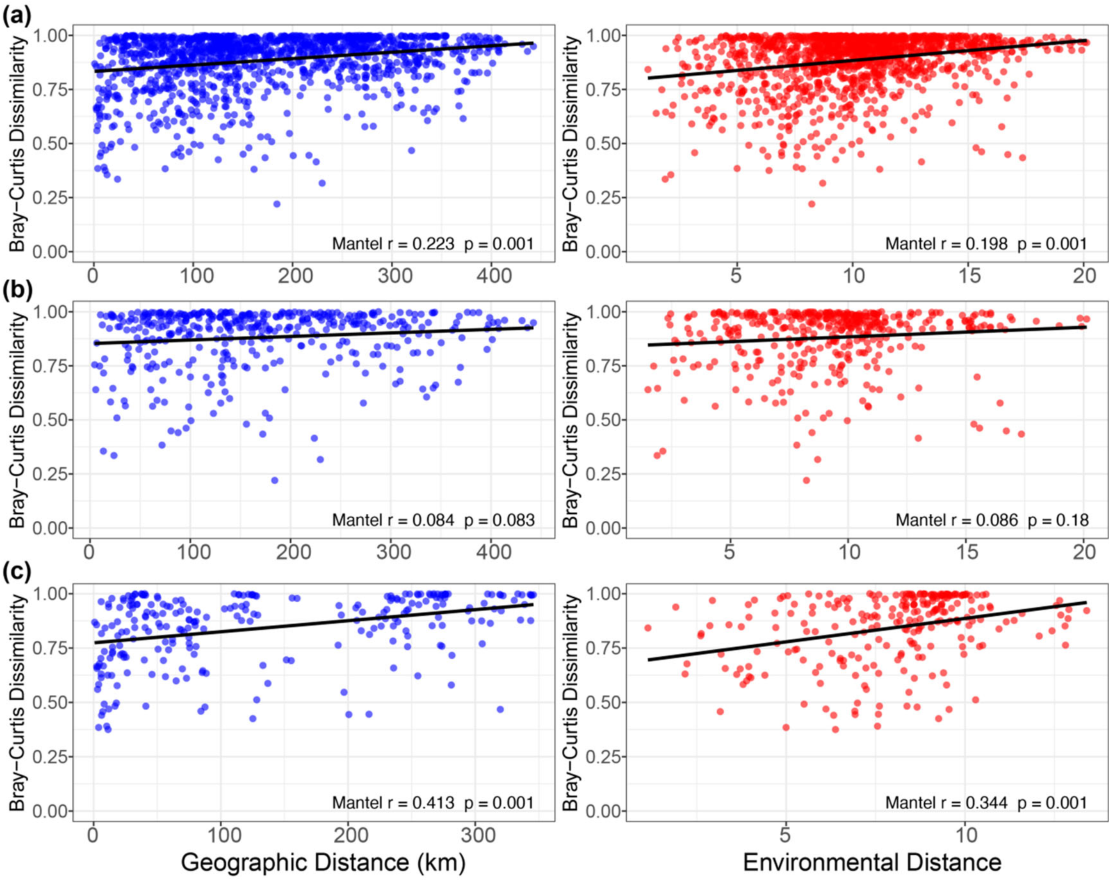

8. Distance attenuation model of Bray-Curtis dissimilarity of protozoan communities along geographic distance and environmental distance. (a): all sampling sites; (b): with non-native fish species; (c): with out non-native fish species.

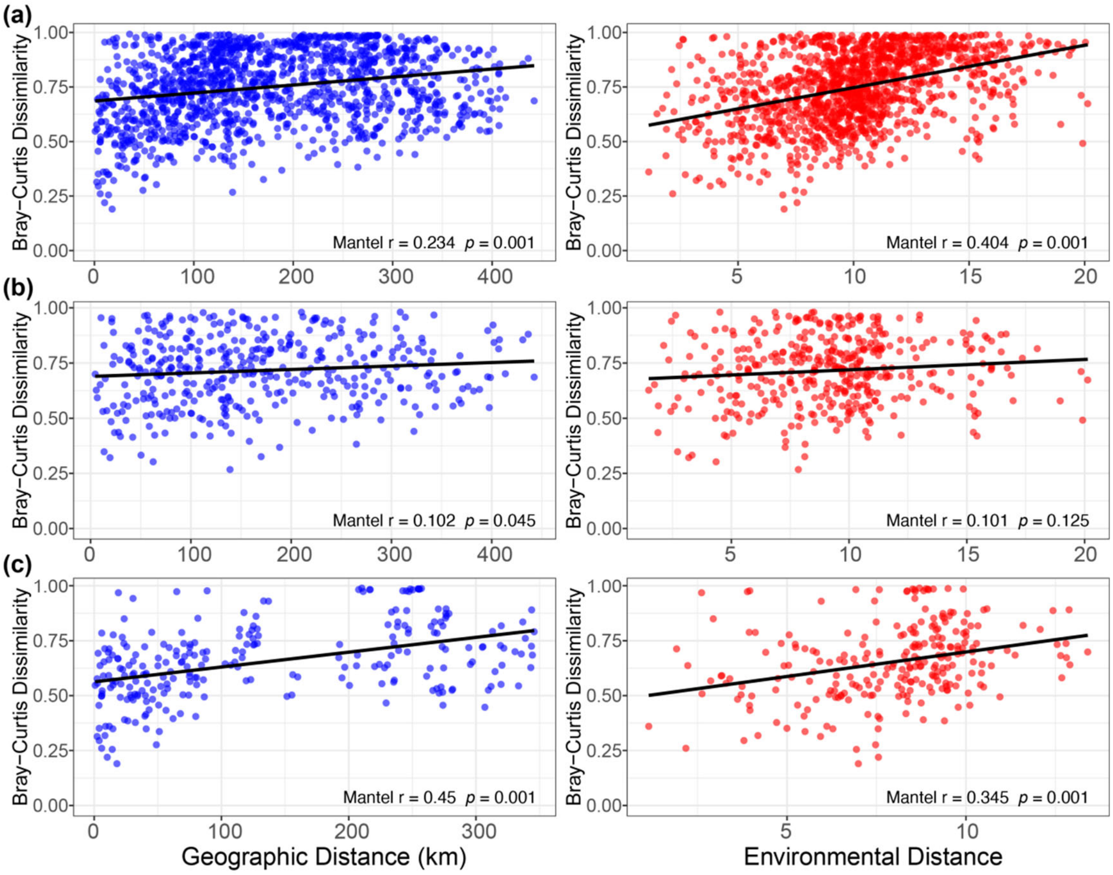

9. Distance attenuation model of Bray-Curtis dissimilarity of fungi communities along geographic distance and environmental distance. (a): all sampling sites; (b): with non-native fish species; (c): with out non-native fish species.

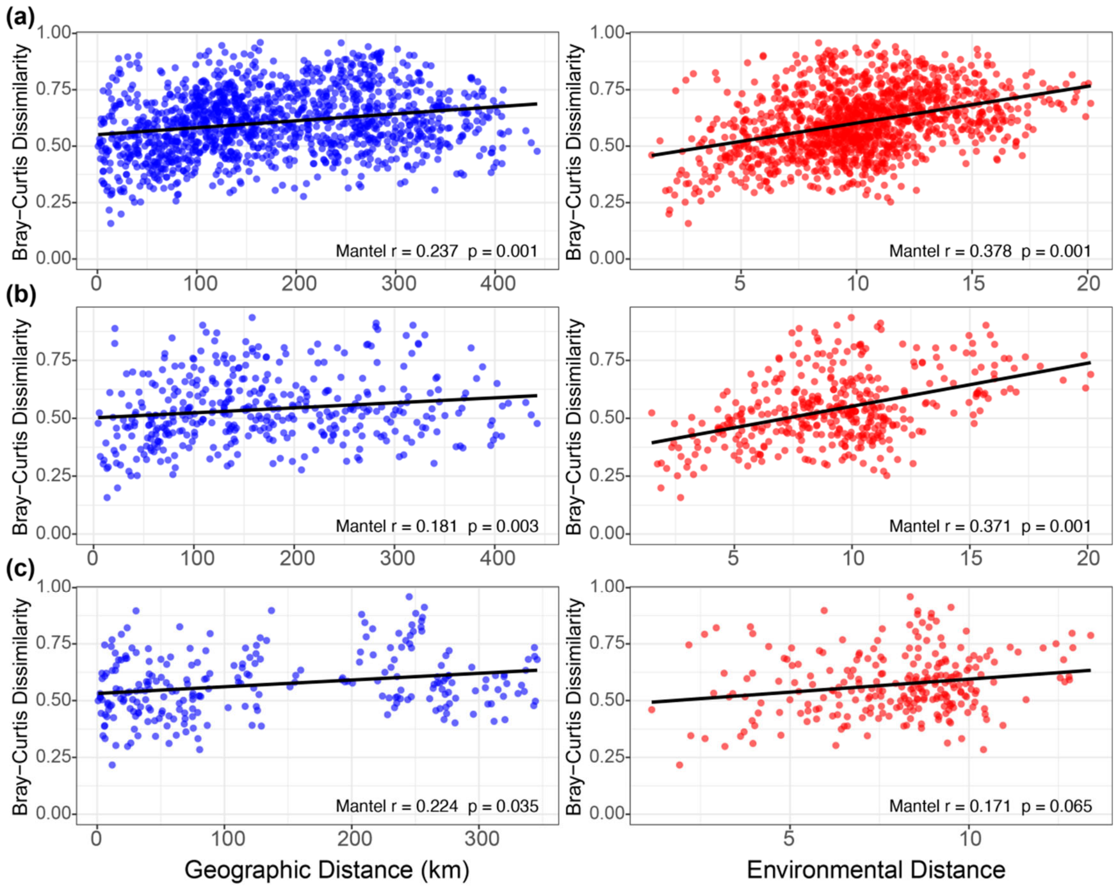

10. Distance attenuation model of Bray-Curtis dissimilarity of heterotrophic communities along geographic distance and environmental distance. (a): all sampling sites; (b): with non-native fish species; (c): with out non-native fish species.

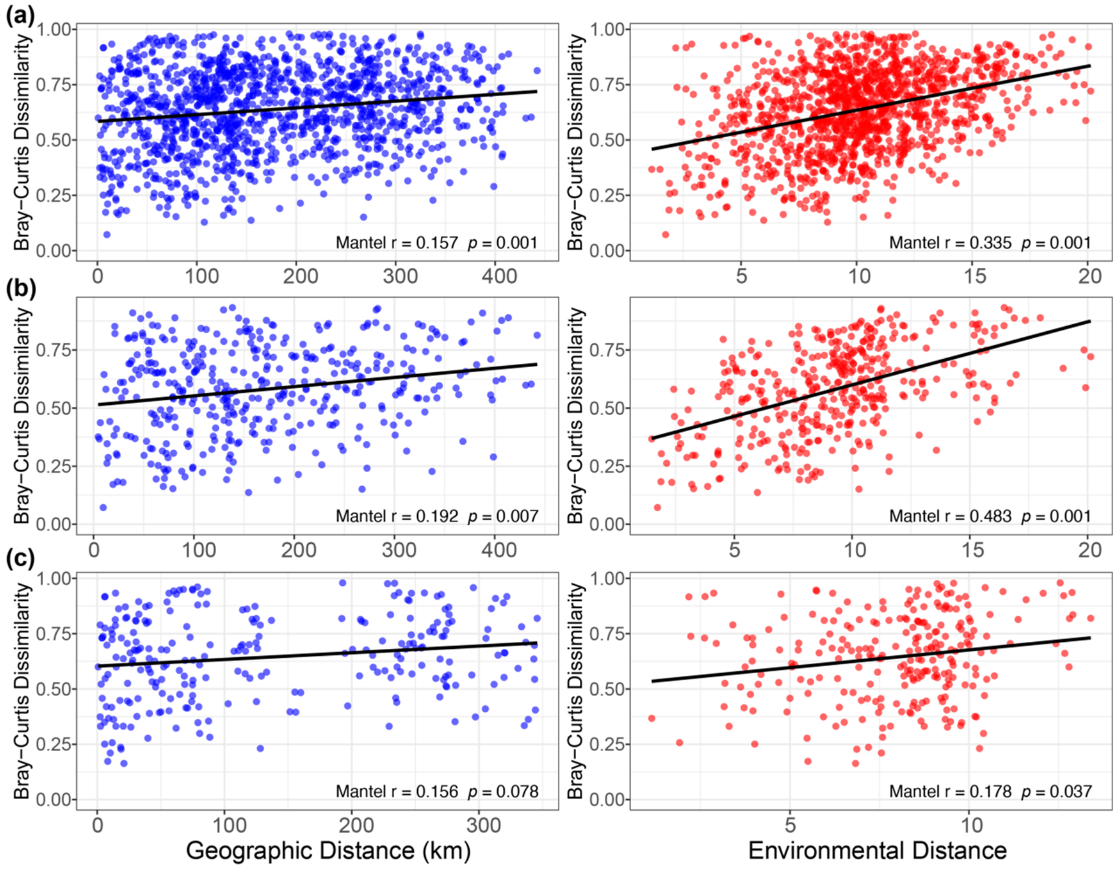

11. Distance attenuation model of Bray-Curtis dissimilarity of autotrophic communities along geographic distance and environmental distance. (a): all sampling sites; (b): with non-native fish species; (c): with out non-native fish species.
